# Recent Advances in Electrochemical and Simulation-Based Methods for Understanding Interfacial Charge Storage Mechanisms in Supercapacitor Materials: Analysis and Perspectives

**DOI:** 10.3390/nano16211370

**Published:** 2026-10-27

**Authors:** Freddy Escobar-Teran, Kleber Andrade

**Affiliations:** Departamento de Ciencias Exactas, Universidad de las Fuerzas Armadas ESPE, Sangolquí 171103, Ecuador; kgandrade@espe.edu.ec

**Keywords:** interfacial charge storage mechanisms, supercapacitors, EQCM (electrochemical quartz crystal microbalance), EIS (electrochemical impedance spectroscopy), ac-electrogravimetry, in situ NMR (nuclear magnetic resonance), computer simulations

## Abstract

The intermittent nature of renewable energy sources requires efficient energy storage technologies to prevent losses. Supercapacitors are key devices for these applications due to their high power density and rapid electrostatic storage capabilities. This review summarizes recent advances in electrochemical and simulation-based methods used to study charge storage at the electrode/electrolyte interface. We examine ion transport kinetics, species identification, and the solvation and desolvation dynamics that govern the electrochemical process. Finally, we provide a critical assessment of current analytical methodologies and outline future perspectives, emphasizing that the synergy between computational methods and experimental techniques will drive the development of next-generation supercapacitors with enhanced performance.

## 1. Introduction

Reducing the consumption of fossil fuels is crucial to mitigating greenhouse gas emissions. Climate change driven by conventional energy sources requires an urgent transition toward renewable and environmentally sustainable energy sources [1,2,3]. To achieve this goal, more efficient electrical energy storage devices are essential [4]. Batteries and supercapacitors are the most commonly used electrochemical energy storage technologies. Batteries store energy through faradaic redox reactions, providing high-energy delivery with energy densities reaching a few hundred Wh kg^−1^. However, these battery-type faradaic reactions undergo slow kinetics, leading to limited power performance and cycle life [5]. In contrast, supercapacitors represent a promising energy storage solution due to their high-power density, long cycle life, excellent stability, and safety. Furthermore, their charge storage processes are fast and highly reversible [6,7]. Supercapacitors can be classified into two types: (i) electrochemical double-layer capacitors (EDLCs) and (ii) pseudocapacitors [8]. The capacitance of EDLCs is due to the electrostatic interaction at the electrode/electrolyte interface. Consequently, EDLCs can achieve fast charging/discharging rates and good cycling stability. However, the energy density of EDLCs is limited due to their surface electrostatic mechanism [9]. Conversely, pseudocapacitors store charge via fast and reversible faradaic redox reactions occurring at or near the surface of active materials. Thus, pseudocapacitors can exhibit a much larger capacitance than EDLCs [10]. Although traditional transition metal oxides (e.g., MnO_2_) often suffer from low intrinsic electronic conductivity [11], advanced pseudocapacitive materials—most notably MXenes (Ti_3_C_2_T_x_)—combine fast surface redox kinetics with metallic-like electronic conductivity, delivering both high energy density and superior power performance without kinetic bottlenecks [12,13]. As mentioned, energy and power density are crucial considerations when selecting or designing a supercapacitor.

To avoid common misclassifications in the literature, a clear distinction must be made between pseudocapacitive materials and battery-type faradaic materials [11,14]. While both rely on faradaic electron transfer, their thermodynamic, kinetic, and structural mechanisms are fundamentally different:

- Pseudocapacitive Storage: This occurs via fast, highly reversible faradaic reactions at or near the surface without phase transitions [10,11]. It exhibits capacitive electrochemical signatures, including a continuous charge-potential relationship (linear *Q* vs. *V*), quasi-rectangular cyclic voltammetry (CV) profiles, and a current response that scales linearly with the scan rate (*v*). Quantitatively, this is evaluated using the power-law relationship *i* = *av^b^* (where *i* is the peak current, *v* is the scan rate, and *a* and *b* are adjustable parameters). A value of *b* ≈ 1 (determined from the slope of log(*i*) vs. log(*v*)) signifies surface- or near-surface-controlled capacitive kinetics [10,12]. This encompasses both intrinsic pseudocapacitors (e.g., RuO_2_, MnO_2_) and extrinsic/intercalation pseudocapacitors (e.g., Nb_2_O_5_, MXenes), where ions intercalate into 2D layers without phase changes [12,14].
- Battery-Type Faradaic Storage: Relies on semi-infinite, diffusion-controlled redox reactions involving distinct phase transitions within the bulk material (e.g., transition metal hydroxides like Ni(OH)_2_ and Co(OH)_2_, or conventional battery intercalators) [11,15]. These materials exhibit sharp, widely separated oxidation/reduction peaks in CVs, non-linear galvanostatic charge–discharge profiles with distinct voltage plateaus, and a power-law factor *b* ≈ 0.5 [10,15]. Therefore, redox-active oxides or hydroxides showing diffusion-limited kinetics and phase transformations should be classified as battery-type electrodes rather than pseudocapacitors, regardless of their reversibility or application in hybrid devices [11].

Beyond understanding these fundamental storage mechanisms, integrating pseudocapacitive materials into compact architecture is essential for practical micro-electronics. Miniaturizing energy storage devices on a chip can significantly enhance their areal and volumetric performance metrics, although gravimetric energy density (Wh kg^−1^) remains governed by the active material’s intrinsic properties. Micro-supercapacitors (MSCs) typically feature interdigitated planar architectures with metallic current collectors and finger electrodes separated by an insulating gap [16]. The electrolyte is subsequently coated on top of the device to facilitate ion transport across the electrodes. The total footprint of micro-supercapacitors ranges from a few microns to centimeters [16,17]. At these micro-scales, the short ion diffusion distance in the electrolyte and the exposed-side edges of the microelectrodes enable rapid electrolyte infiltration and highly efficient utilization of the active surface area. Consequently, this architecture leads to a marked increase in both areal (mWh cm^−2^) and volumetric (mWh cm^−3^) energy and power densities [9,18]. However, a realistic assessment of these miniaturized systems requires accounting for non-active components (such as substrates, current collectors, and packaging materials), which constitute a major fraction of the overall device mass and volume [19]. When properly optimized, MSCs can be seamlessly integrated onto single chips, making them ideal compatible micro-power sources for smart and wearable electronics.

Carbon-based materials are widely employed as electrode materials for supercapacitors. The relationship between the pore size and the ion size is a critical factor governing specific capacity [20]. However, determining the precise interfacial charging mechanisms remains experimentally challenging, as conventional electrochemical or physical characterizations often lack the spatial or temporal resolution required.

In situ small-angle scattering (SAS), in situ X-ray diffraction (XRD), and infrared spectroelectrochemistry have provided structural and mass-transfer insights. However, capturing the fast dynamic aspects of ion electroadsorption and enabling molecular-level ion counting within complex carbon electrodes remains a major challenge. To access a more comprehensive description of the electrical double layer (EDL), EQCM has also been combined with electronic conductance studies or nuclear magnetic resonance (NMR) spectroscopy [21,22,23]. Furthermore, to overcome these constraints, coupled techniques such as ac-electrogravimetry (coupling fast QCM with electrochemical impedance spectroscopy) have emerged to isolate charged from uncharged species and determine kinetic transfer constants [6,20,24,25,26]. Finally, combining these advanced in situ experimental techniques with computational modeling methods—such as molecular dynamics (MD), Monte Carlo (MC) simulations, and density functional theory (DFT)—provides a deeper, atomistic understanding of interfacial charge storage mechanisms [27].

In this context, this review summarizes recent progress in both electrochemical techniques and computational modeling for determining interfacial charge storage mechanisms. Furthermore, we critically assess current analytical methodologies and propose integrated experimental–computational frameworks to guide the design of the next-generation, high-performance supercapacitors.

### Principle of Operation for Supercapacitors

EDLCs typically consist of two electrodes immersed in an electrolyte, with a separator positioned between them [2]. Active carbon, CNTs, and graphene are commonly used as electrode materials due to their large specific surface area, high electrical conductivity, and electrochemical conductivity [9]. Furthermore, the choice of electrode material plays an important role in determining the overall performance of supercapacitors [25]. Compared with conventional capacitors, supercapacitors can store much more energy, mainly because their electrode materials have a very large specific surface area [2]. In EDLCs, this energy-storage process is mainly associated with the reversible accumulation of electrical charges at the electrode/electrolyte interface, resulting in the formation of an electrochemical double layer [28] (Figure 1). In other words, no redox reaction is involved in the electrochemical process. When a supercapacitor is subjected to a potential difference between two electrodes, ions in the electrolyte will move toward the surface of the electrode of opposite polarity. Thus, charges are accumulated and form two loaded layers at the electrode/electrolyte interface [9,29]. The accumulation of ionic species at the electrode/electrolyte interface can be described as two capacitors in series (C_1_ and C_2_). Thus, the total capacitance of the cell (C) can be estimated according to the following equation [28]:

(1) $$\frac{1}{C}=\frac{1}{C_{1}}+\frac{1}{C_{2}}$$

In contrast to EDLCs, pseudocapacitors store charge through rapid and reversible redox reactions occurring at the electrode/electrolyte interface (Figure 1). The ions can enter the near-surface region or intercalate between layers to participate in faradaic charge transfer. Consequently, pseudocapacitors can achieve substantially higher specific capacitance than EDLCs under comparable testing conditions [18]. While carbon-based EDLCs are physically limited by electrostatic accumulation (typically delivering 100–200 F g^−1^, based on 10–20 μF cm^−2^ of accessible area), pseudocapacitive materials can achieve a 2–5-fold increase in gravimetric capacitance (>400–500 F g^−1^) and up to an order of magnitude higher volumetric capacitance (>1500 F cm^−3^ for Ti_3_C_2_T*_x_* MXene hydrogels) [12]. Mechanistically, pseudocapacitive behavior is broadly categorized into two types: (i) surface redox pseudocapacitance, where charge transfer occurs via surface chemical adsorption/desorption of ions without bulk penetration, and (ii) intercalation pseudocapacitance, where ions rapidly intercalate into crystallographic channels or 2D interlayer spaces without causing a crystallographic phase transition [29,30].

The most common electrode materials for pseudocapacitors are poly(3,4-ethylene dioxythiophene) (PEDOT) [31], polypyrrole (PPy) [32], polyaniline (PANI) [33], and polythiophene (PTh) [34]. Furthermore, transitional metal oxides/hydroxides such as ruthenium oxide (RuO_2_) [35], MnO_2_ [36], ZnO [37], NiO [38], and Co(OH)_2_ [9] have also been widely studied. The basic equation for their reaction processes can be expressed as follows [9]:

M^n+^ + xe^−^ ↔ M^(n−x)+^ (2)

Equation (2) describes a reduction–oxidation process. In this reaction, M represents a metallic catión with a positive charge of magnitude *n*, while *e* denotes the electrons transferred. Meanwhile, *x* corresponds to the number of electrons gained by the metallic ion in order to be converted into a different metallic species.

A representative example of this mechanism in transition metal hydroxides is the nickel hydroxide/nickel oxyhydroxide (Ni(OH)_2_/NiOOH) redox couple, which operates via coupled proton–electron transfer:

Ni(OH)_2_ ↔ NiOOH + H^+^ + e^−^ (3)

## 2. Diagnostic Tools for Explaining Charge Storage Mechanisms in Supercapacitor Materials

Understanding charge storage mechanisms in supercapacitor materials requires robust analytical tools. This section discusses several characterization methods, including electrochemical impedance spectroscopy (EIS), electrochemical quartz crystal microbalance (EQCM), ac-electrogravimetry, nuclear magnetic resonance (NMR) spectroscopy, and computational simulations.

### 2.1. Electrochemical Impedance Spectroscopy (EIS)

Electrochemical impedance spectroscopy (EIS) serves as a non-invasive diagnostic in the frequency domain, capable of resolving overlapping interfacial phenomena—such as bulk electrolyte resistance, charge-transfer kinetics, and diffusional polarization—spanning multiple frequency ranges [39,40]. Electrochemical reactions often proceed through a sequence of elementary steps with distinct kinetic profiles. Resolving these individual steps is essential for mapping reaction mechanisms, yet conventional electroanalytical techniques frequently fail to differentiate them. EIS overcomes this limitation by decoupling processes across their characteristic timescales, making it a standard tool for probing interfacial phenomena [41,42].

EIS operates by applying a small-amplitude sinusoidal potential perturbation (Δ*V*) around a steady-state bias (*V_s_*). Modulating the perturbation across a broad frequency range separates the system’s kinetic responses: rapid processes react at high frequencies, while slower mechanics emerge at lower frequencies [43,44,45,46]. Although the global current–voltage (*I* − *V*) relationship of an electrochemical system is non-linear, constraining the modulation to a tiny segment of the Lissajous curve satisfies local linearity. The resulting current response (Δ*I*) thus remains sinusoidal, oscillating around a stationary Iseline (*I_s_*) and enabling the generalization of electrical resistance into complex impedance via Fourier analysis (see Figure 2).

The transfer function ∆*V*/∆*I*(ω), called the electrochemical impedance, can be defined with *ω =* 2*πf,* corresponding to the pulsation.

The electrochemical impedance *Z*(*ω*) is a complex number that can be represented either in polar coordinates (|*Z*|,φ*ϕ*) or Cartesian coordinates (*Re Z*, *Im Z*):

*Z*(*ω*) = |*Z*|*exp j ϕ* = *Re Z* + *j Im Z* (4)

The relationship between these quantities is:

On the one hand,

|*Z*|^2^ = (*Re Z*)^2^ + (*Im Z*)^2^ (5)

(6) ϕ=arctanImZReZ

On the other hand,

*Re Z* = |*Z*| *cos ϕ* (7)

*Im Z* = |*Z*| *sin ϕ* (8)

where |*Z*| is the impedance modulus, the ratio of amplitudes between ∆*V* and ∆*I*; *Re Z* and *Im Z* are the real and imaginary parts of *Z*(*ω*); Φ*ϕ* is the phase angle between ∆*V* and ∆*I;* and *j* is the complex number (*j*^2^ = −1).

A Nyquist plot is commonly employed in supercapacitors and pseudocapacitors to evaluate three distinct frequency-dependent regions: a high-frequency semicircle, a mid-frequency Warburg-type impedance transition (≈45^∘^), and a steep low-frequency vertical tail. These features correspond to distinct electrochemical processes across the frequency domain, namely electrolyte and contact resistance (*R_s_*) at very high frequencies, charge-transfer resistance (*R_ct_*) coupled with double-layer capacitance (*C_dl_*) at high-to-intermediate frequencies, porous ion diffusion (*W*) at mid-frequencies, and capacitive charge accumulation at low frequencies. The foundational model for these systems is adapted from the Randles equivalent circuit [48,49,50].

As illustrated in Figure 3, a hybrid supercapacitor model incorporates the solution resistance (*R_s_*), the charge-transfer resistance (*R_ct_*), the double-layer capacitance (*C_dl_* or non-ideal *CPE_DL_*), the Warburg diffusion impedance (*W*), and a low-frequency capacitive element (*C_edl_*).

To guarantee the physical validity and interpretability of EIS spectra in supercapacitive systems, the experimental data must satisfy three core criteria: linearity, stationarity, and causality [52]. Linearity is experimentally preserved by restricting the AC perturbation amplitude to the small-signal regime (Δ*E* ≤ 10 mV), whereas stationarity requires that the electrode–electrolyte system remains time-invariant throughout the frequency sweep [53]. The compliance of experimental spectra with these fundamental criteria should be rigorously validated using Kramers–Kronig (K–K) integral transforms prior to equivalent circuit fitting [53,54].

Furthermore, while classical Randles-type models treat the interface as a planar boundary, real nanostructured electrodes exhibit complex porous architectures [55]. Consequently, Porous-Electrode Transmission Line Models (TLMs), originating from de Levie’s foundational theory [55], provide a physically accurate description. These models account for the distributed electrolyte resistance along the pore depth (*r_m_*) in parallel with the interfacial double-layer capacitance or pseudocapacitive charge transfer (*c_m_*), naturally explaining the characteristic 45^∘^ Warburg-like region at intermediate frequencies and its transition to purely capacitive behavior at low frequencies [55,56].

Electrochemical impedance spectroscopy (EIS) is a widely employed technique for investigating the electrochemical interfacial processes of supercapacitor electrodes [57,58]. For instance, EIS was used to assess the influence of a carbonaceous coating on interfacial charge-transfer processes in PANI and PPy nanowires coated with an approximately 5 nm thick carbon shell, denoted as PANI@C and PPy@C electrodes, respectively [33]. These findings indicate that the *R_s_* of the modified electrodes is higher than that of the bare polymer electrodes, owing to additional contact resistance between the carbonaceous shell and the polymer core.

EIS was also used to study the capacitive behavior of activated carbon (AC) materials in different electrolytes [59]. A quasi-vertical line appeared at low frequencies in all electrolytes, indicating capacitive behavior. Additionally, a semicircle was observed at mid-frequencies, indicating charge-transfer resistance within the electrode. These findings suggest that the resistance in the transition region is attributed to ion diffusion and transport at the electrode/electrolyte interface.

Recently, Anjum et al. [60] used EIS to investigate the charge storage mechanisms of a Ti_3_C_2_Tx MXene working electrode using an Ag/AgCl (saturated KCl) reference electrode. The authors applied different bias voltages in both the anodic and cathodic regions, providing detailed insights in the ranges (Figure 4). In the cathodic potential range, the Nyquist plots exhibit a similar profile (Figure 4a), but the Warburg region shifts to the right. This shift indicates an increase in diffusion resistance as the applied potential becomes increasingly negative.

Figure 4b displays the Nyquist plots recorded at different anodic bias potentials. At 0, 0.2, and 0.25 V, the impedance spectra show a single semicircle followed by a sloping line. When the potential reaches 0.3 V, a second semicircle emerges and becomes increasingly noticeable as the applied potential is raised. This feature in the low-frequency region is likely related to adsorption and desorption processes occurring at the electrode/electrolyte interface. Its appearance at 0.3 V may indicate the adsorption of water molecules and the early stages of oxide-layer formation. At potentials above 0.4 V, the first semicircle increases markedly in diameter, indicating a rise in polarization resistance. According to the authors, this behavior can be attributed to oxide-layer formation, which suppresses Faradaic currents and consequently limits further electrochemical processes within the investigated frequency range.

### 2.2. Electrochemical Quartz Crystal Microbalance (EQCM)

EQCM is a coupled technique that combines Quartz Crystal Microbalance (QCM) with potentiodynamic electrochemical measurements [61,62]. EQCM is composed of a thin piezoelectric quartz crystal sandwiched between two metal electrodes (e.g., gold electrodes) used to create an alternating electric field across the crystal, causing vibrational motion of the crystal at its resonance frequency. In other words, when an alternating voltage is applied between a pair of gold electrodes, the upper and the lower faces of the active zone resonate (parallel to each other). A stationary acoustic wave is generated throughout the quartz crystal. The frequency of this wave is called the resonant frequency of the quartz resonator [63,64] (See Figure 5).

The shift in the normalized resonant frequency (Δ*f/n*) can be converted into an areal mass change (Δ*m/A*) or total mass change (Δ*m)* on the crystal surface by applying Sauerbrey’s equation [65]:

(9) $$\Delta f_{n}=-\left(\frac{2nf_{o}^{2}}{A\sqrt{\rho_{q}\mu_{q}}}\right)\Delta m=-nC_{f}\left(\frac{∆m}{A}\right)$$

where *ρ_q_* is the quartz density (2.648 g cm^−3^); *μ_q_* represents the shear modulus of the AT-cut quartz crystal, with a value of 2.947 × 10^11^ g cm^−1^ s^−2^; *f*_0_ denotes the fundamental resonant frequency of the quartz crystal (9 MHz); *A* corresponds to the active area of the quartz surface covered by the deposited metal electrode (cm^2^); and *n* is the overtone number (*n* = 1, 3, 5 …).

The theoretical Sauerbrey sensitivity constant ($C_{f}=\frac{2f_{0}^{2}}{\sqrt{\rho_{q}\mu_{q}}}$) for a 9 MHz crystal is 18.3 × 10^7^ Hz g^−1^cm^2^ [66]. Expressed per unit area, the normalized frequency change relates directly to the areal mass change (Δ*m_areal_* = Δ*m*/*A*, in g cm^−2^):

(10) $$\frac{\Delta f_{n}}{n}=-C_{f}\Delta m_{\mathrm{areal}}$$

EQCM is a powerful technique for monitoring the electrode/electrolyte interface because it can detect mass changes in the order of 1 nanogram [67], and the simultaneous measurement of mass and charge changes during electrode cycling enables the determination of the overall mass per mole of electrons (MPE) transferred between the electrode and the electrolyte. When only a single species participates in the exchange, the MPE directly corresponds to its molar mass. However, when several ionic species are involved, the interpretation of conventional EQCM data using Faraday’s law becomes limited [68]. To better distinguish and identify the species responsible for charge compensation, Donnan-type electrical double layer models have been integrated into the gravimetric equations of EQCM [69]. Furthermore, the complex nature of electrode mass variations, together with the viscoelastic behavior of the electrode material and any potentially formed solid electrolyte interface (SEI) layer, can be explored through multiharmonic EQCM with dissipation monitoring (EQCM-D) [70,71,72,73]. This approach provides valuable insight into how factors such as electrolyte composition, ionic species, and binders influence structural changes in the electrodes [74].

Examining the literature, a certain number of papers employing EQCM to study carbon materials for supercapacitors have been reported in recent years. In 2009, Levi et al. [75] applied EQCM to monitor ion flux in porous carbon materials, thereby introducing this advanced in situ technique for analyzing supercapacitor charging mechanisms. Subsequently, EQCM was employed to study ion transport and desolvation processes in supercapacitors by comparing theoretical mass changes with experimentally measured variations [21,76,77]. In 2014, EQCM was used to characterize ion adsorption in carbide-derived carbon (CDC) with two different average pore sizes (1 and 0.65 nm), from neat and solvated 1-Ethyl-3-methylimidazolium bis(trifluoromethanesulfonyl) imide (EMI-TFSI) electrolytes. The findings indicate that for CDCs with ~1 nm pores—comparable to the size of both cations and anions—charge compensation during negative polarization is governed exclusively by cations. In contrast, under positive polarization, an ion-exchange mechanism (between anions and cations) dominates at low charge densities, whereas counterion (anion) adsorption becomes predominant at higher charge densities [78].

Using EQCM, a 2016 study by Escobar-Teran et al. [20] investigated the electrochemical performance of SWCNT-based thin films in 0.5 M aqueous NaCl (Figure 6). The observed cyclic voltammetry profile exhibits a quasi-rectangular shape (Figure 6A), signaling that ion electroadsorption drives the reversible charge storage mechanism.

In parallel, real-time mass tracking via QCM (Figure 6B) confirms that species transfer shifts from cations at negative potentials to anions at positive potentials. Furthermore, the change in slope around 0.1 V vs. Ag/AgCl corresponds to the point of zero charge (PZC). To identify the nature of the species being transferred, the $F\times \frac{∆m}{∆q}=F\frac{∆m/∆t}{∆q/∆t}$ function was calculated from the EQCM data (Figure 6A,B). This relationship corresponds to the molar mass of the species exchanged at the electrochemical interface for a given scan rate.

In 2017, Gouba et al. used the EQCM technique to study the capacitive charge storage behavior of the ERGO (Electrochemical Reduced Graphene Oxide) electrodes. The authors evaluated carbon electrodes in several aqueous electrolytes, including LiCl, KCl, and NaCl (Figure 7). They found that the mass changes recorded in LiCl and KCl electrolytes were nearly identical (Figure 7B), while the corresponding current responses were also very similar (see Figure 7A). The authors attribute this behavior to equal charge adsorption at the electrode–electrolyte interface. Yet this explanation falls short once one factors in ionic molecular weight, especially if only cations are assumed to drive the process. To clarify this, they point out that Li^+^ ions actually drag along free solvent molecules during electroadsorption, an effect far more pronounced here than in aqueous KCl systems.

Additionally, both Li^+^ and K^+^ species are electroadsorbed in their hydrated forms, although they differ in their respective degrees of hydration.

In 2018, Lé et al. [80] investigated the energy storage mechanisms in vertically oriented graphene nanosheet (VOGN) supercapacitor electrodes using the EQCM technique. The carbon electrodes were tested in a solution of propylene carbonate (PC) containing 0.5 M TBABF4. The EQCM technique provided key insights into the ion-exchange mechanisms between VOGNs and organic electrolytes, revealing that anions play a dominant role.

The EQCM technique was also used to study the charge storage properties of SWCNT/Prussian blue nanocube composites [24]. The EQCM results reveal a distinctive relationship between mass variation and current response, which can be divided into three regions (Figure 8). Zone I is attributed primarily to the contribution of cations, while Zone III is dominated by anions.

These behaviors can be attributed to the adsorption and desorption of each ion as the surface is negatively and positively charged, respectively. Zone II presents a different behavior, characterized by a decrease in mass during the anodic-to-cathodic scan, followed by an increase in mass during the sweep back.

The authors suggest that this behavior is due to a cooperative effect between the SWCNT and PB networks that shapes the net mass response.

In 2021, the capacitive properties of nanocomposite carbon film electrodes were also examined by Escobar-Teran et al. [26]. Using the EQCM technique, the electrochemical behavior of SWCNT/PPy composite electrodes was analyzed in a 0.5 M aqueous NaCl electrolyte (Figure 9). The results showed that the PPy component contributes only slightly to the cyclic voltammetry (CV) response (Figure 9a) when compared to pristine SWCNTs [20]. However, a marked difference in both amplitude and profile is evident in the mass response of the carbon film relative to SWCNTs alone (Figure 9b). These qualitative findings indicate that the incorporation of PPy modifies the interfacial ion-exchange dynamics of the SWCNT electrodes.

In 2022, Zhang et al. [81] demonstrated, at a molecular level, that N-doping strongly influences the charging mechanism in porous carbon with the aid of EQCM.

Using EQCM, Turgeman et al. [82] confirmed that pseudocapacitive charge storage in polyaniline (PANI) organic electrodes involves coupled ion uptake/release and solvent molecule co-transport. The extent of this solvation co-transport was shown to be strongly governed by the structural pore architecture and mechanical rigidity of the PANI matrix.

In 2025, Li et al. [83] used the EQCM technique to explain multi-stage charge storage on nanoporous carbons in Zn-ion hybrid capacitors. The findings revealed a four-stage charge–storage process under cathodic polarization, comprising adsorption and desolvation of hydrated Zn^2+^ ions, followed by water reduction, catalysis by Zn^2+^, and the formation of adsorbed hydrogen atoms (H_ad_) (Figure 10).

In the same year, Bouzina et al. [84] investigated the charge compensation mechanism of a graphene–polydopamine electrode in the presence of various alkali metal sulfate aqueous electrolytes (Li_2_SO_4_, Na_2_SO_4_, and K_2_SO_4_). For this purpose, the EQCM technique was used, which is shown in Figure 11. According to the authors, the voltammograms have almost rectangular shapes (Figure 11A–C), which is characteristic of double-layer capacitance. With respect to the nature of the alkali metal electrolyte, a non-selective ion adsorption behavior is observed, suggesting that the charge storage mechanism is predominantly capacitive and primarily controlled by the accessible surface area rather than specific chemical interactions. Additionally, the EQCM-derived mass profiles (Figure 11D–F) exhibit a steady increase during the cathodic scan and a corresponding decrease during the anodic scan for all electrolytes.

This behavior indicates that cations play a dominant role in preserving electroneutrality throughout the charge–discharge process.

Using EQCM, Yip et al. [85] elucidated a co-ion desorption mechanism in a Ti_3_C_2_T*_x_* MXene/conjugated polymer electrolyte asymmetric pseudocapacitor. The process is governed by sulfonate side-group charge compensation and cation expulsion during oxidation (charging), followed by cation re-ingression during reduction (discharging) to preserve electroneutrality.

Nambiar et al. [86] also demonstrated, via EQCM, that the electrolyte pH governs ion-adsorption selectivity on functionalized carbon materials. Specifically, passing the isoelectric point (pI) triggers a shift from anion- to cation-dominated charge storage, confirming that surface functional group ionization controls selective ion uptake.

These applications highlight the key role of the EQCM in investigating the charging mechanisms of supercapacitor electrodes.

### 2.3. Ac-Electrogravimetry

Ac-electrogravimetry has proven to be an attractive tool for studying the nature of the ionic flux at the electrode/electrolyte interface. This technique was first proposed by Gabrielli et al. [87] at the LISE laboratory in France. Ac-electrogravimetry is a coupling technique between fast quartz crystal microbalance (QCM) measurements and electrochemical impedance spectroscopy (EIS), allowing changes in both mass and electrochemical response to be monitored simultaneously. The basic principle is similar to that of electrochemical impedance measurements described in the previous section. In this technique, a small sinusoidal voltage signal (ΔE) is superimposed on a constant potential and applied between the reference and working electrodes. This perturbation produces small changes in the mass of the film deposited on the quartz surface, which can then be used to determine the electrogravimetric transfer function.

When the gold electrode of the quartz resonator is used as the working electrode, two different transfer functions can be obtained at the same time: the electrochemical impedance $\frac{\Delta E}{\Delta I}\left(\omega \right)$ and an electrogravimetric or mass/potential transfer function $\frac{\Delta m}{\Delta E}\left(\omega \right)$, as illustrated in Figure 12 [88,89,90].

This technique provides valuable information on several aspects of the electrode/electrolyte interface, including (i) the identification of the species involved and the direction of their fluxes, (ii) the distinction between charged and neutral species, (iii) the assessment of their transport kinetics, and (iv) changes in their relative concentrations [87,91,92,93].

In this technique, the electroadsorption/desorption mechanism that intervenes in the electrochemical process is as follows:

(11) $$\mathrm{Cation}:\left\langle P\right\rangle +e^{-}+C^{+}\;\overset{K_{c1}K_{c2}}{⇄}\;\left\langle P,C^{+}\right\rangle$$

(12) $$\mathrm{Anion}:\left\langle P\right\rangle +A^{-}\;\overset{K_{a1}K_{a2}}{⇄}\;\left\langle P,A^{-}\right\rangle +e^{-}$$

where <P> is the free site “in” the film, <P,C^+^> is the site occupied with a monovalent cation, and <P,A^−^> is the site occupied with a monovalent anion. The *K_i_* values are related to the transfer kinetic rates. Then, the instantaneous flux of each species, *J_i_*, can be written as follows*:*

For a cation:

(13) $$J_{c}=-d_{f}\left(K_{c1}\left(C_{C^{+},\max}-C_{C^{+}}\right){\left[C^{+}\right]}_{\mathrm{sol}}-K_{c2}\left(C_{C^{+}}-C_{C^{+}\min}\right)\right)$$

For an anion:

(14) $$J_{c}=-d_{f}\left(K_{a1}\left(C_{A^{-},\max}-C_{A^{-}}\right){\left[A^{-}\right]}_{\mathrm{sol}}-K_{a2}\left(C_{A^{-}}-C_{A^{-}\min}\right)\right)$$

where *C_i_*_,*max*_ is the maximum concentration of P sites occupied in the film by cations or anions, *C_i_* is the concentration in the film of cations or anions associated with the P sites, and *C_i_*_,*min*_ is the minimum concentration of P sites occupied in the film by cations or anions.

Under the effect of a sinusoidal potential perturbation, Δ*E*, imposed on the film/electrolyte interfaces, sinusoidal concentration fluctuations, Δ*C_i_*, and the flux, Δ*J_i_*, are observed (a: anion, c: cation, s: solvent) in the following form:

(15) $$\Delta J_{i}=-d_{f}\frac{d(\Delta C_{i})}{\mathrm{dt}}$$

where *d_f_* is the film thickness (m), Δ*J_i_* is the variation in the flux related to species i crossing the film/electrolyte interface (mol.m^−2^.s^−1^), and Δ*C_i_* is the variation in the concentration in the film (mol.m^−3^).

In the dynamic regime, the flux change *i* is a function of the potential E, and the concentration of the species, Δ*C_i_*, can also be written as:

(16) $$\Delta J_{i}=\frac{\partial J_{i}}{\partial C_{i}}\Delta C_{i}+\frac{\partial J_{i}}{\partial E}\Delta E=K_{i}\Delta C_{i}+G_{i}\Delta E$$

Then, the ${\left.\frac{\Delta C_{i}}{\Delta E}\right|}_{\mathrm{th}}\left(\omega \right)$ transfer function can be calculated using Equations (13) and (14):

(17) $${\left.\frac{\Delta C_{i}}{\Delta E}\right|}_{\mathrm{th}}\left(\omega \right)=\frac{-G_{i}}{\mathrm{j\omega}d_{f}+K_{i}}$$

where *ω =* 2*πƒ* is the pulsation, ƒ is the frequency of potential modulation, and *K_i_* and G_i_ are the partial derivatives of the flux with respect to the concentration and the potential, respectively. The relationships are as follows: $K_{i}={\left(\frac{\partial J_{i}}{\partial C_{i}}\right)}_{E}$ and $G_{i}={\left(\frac{\partial J_{i}}{\partial E}\right)}_{C_{i}}$.

Furthermore, *K_i_* corresponds to the kinetics of the ionic or free solvent transfer, while Gi corresponds to the inverse of the transfer resistance, $R_{t_{i}}=\;\frac{1}{FG_{i}}$, of the species at the electrode/electrolyte interface.

The charge/potential transfer function, ${\left.\frac{\Delta q}{\Delta E}\right|}_{\mathrm{th}}\left(\omega \right)$, calculated for the adsorption/desorption of two cations, *c1* and *c2*, and an anion, a, using the Faraday number, *F*, can be written as follows:

(18) $${\left.\frac{\Delta q}{\Delta E}\right|}_{\mathrm{th}}\left(\omega \right)=-d_{f}F\left(\frac{\Delta C_{c1}}{\Delta E}+\frac{\Delta C_{c2}}{\Delta E}-\frac{\Delta C_{a}}{\Delta E}\right)$$

Replacing Equation (15) with Equation (17) gives:

(19) $${\left.\frac{\Delta q}{\Delta E}\right|}_{\mathrm{th}}\left(\omega \right)=d_{f}F\left(\frac{G_{c1}}{(\mathrm{j\omega}d_{f})+K_{c1}}+\frac{G_{c2}}{(\mathrm{j\omega}d_{f})+K_{c2}}-\frac{G_{a}}{(\mathrm{j\omega}d_{f})+K_{a}}\right)$$

The theoretical electrochemical impedance can be calculated as follows:

(20) $${\left.\frac{\Delta q}{\Delta E}\right|}_{\mathrm{th}}\left(\omega \right)=\frac{1}{\mathrm{j\omega}}\frac{\Delta I}{\Delta E}=d_{f}F\left(-\frac{\Delta C_{c1}}{\Delta E}-\frac{\Delta C_{c2}}{\Delta E}+\frac{\Delta C_{a}}{\Delta E}\right)$$

Replacing Equation (15) with Equation (19) gives:

(21) $${\left.\frac{\Delta E}{\Delta I}\right|}_{\mathrm{th}}\left(\omega \right)=\frac{1}{\mathrm{j\omega}d_{f}F\left(\frac{G_{c1}}{\mathrm{j\omega}d_{f}+K_{c1}}+\frac{G_{c2}}{\mathrm{j\omega}d_{f}+K_{c2}}-\frac{G_{a}}{\mathrm{j\omega}d_{f}+K_{a}}\right)}$$

The theoretical electrogravimetric transfer function, ${\left.\frac{\Delta m}{\Delta E}\right|}_{\mathrm{th}}\left(\omega \right)$, can be calculated, taking into account the charged/uncharged species contribution:

(22) $${\left.\frac{\Delta m}{\Delta E}\right|}_{\mathrm{th}}\left(\omega \right)={-d}_{f}\left(m_{c1}\frac{G_{c1}}{(\mathrm{j\omega}d_{f})+K_{c1}}+m_{c2}\frac{G_{c2}}{(\mathrm{j\omega}d_{f})+K_{c2}}+m_{a}\frac{G_{a}}{(\mathrm{j\omega}d_{f})+K_{a}}+m_{s}\frac{G_{s}}{(\mathrm{j\omega}d_{f})+K_{s}}\right)$$

where *m_c_*_1_, *m_c_*_2_, *m_a_*, and *m_s_* are the atomic weights of the species involved.

From the theoretical overall electrogravimetric transfer function (21), it is possible to calculate the theoretical partial transfer functions by removing the *c*2 contribution, calculating ${\left.\frac{\Delta m}{\Delta E}\right|}_{\mathrm{th}}^{c1\mathrm{as}}\left(\omega \right)$; or the *c*1 contribution, calculating ${\left.\frac{\Delta m}{\Delta E}\right|}_{\mathrm{th}}^{c2\mathrm{as}}\left(\omega \right)$, or the anion contribution, calculating ${\left.\frac{\Delta m}{\Delta E}\right|}_{\mathrm{th}}^{c1c2s}\left(\omega \right)$, as shown in the following equations:

$${\left.\frac{\Delta m}{\Delta E}\right|}_{\mathrm{th}}^{c1\mathrm{as}}\left(\omega \right)=\frac{∆m}{∆E}\left(\omega \right)+m_{c2}\left(-\frac{\Delta C_{c1}}{\Delta E}\left(\omega \right)-\frac{\Delta C_{c2}}{\Delta E}\left(\omega \right)+\frac{\Delta C_{a}}{\Delta E}(\omega )\right)$$

(23) $$={-d}_{f}\left[\left(m_{c1}-m_{c2}\right)\frac{G_{c1}}{(\mathrm{j\omega}d_{f})+K_{c1}}+\left(m_{a}+m_{c2}\right)\frac{G_{a}}{(\mathrm{j\omega}d_{f})+K_{a}}+m_{s}\frac{G_{s}}{(\mathrm{j\omega}d_{f})+K_{s}}\right]$$

$${\left.\frac{\Delta m}{\Delta E}\right|}_{\mathrm{th}}^{c2\mathrm{as}}\left(\omega \right)=\frac{∆m}{∆E}\left(\omega \right)+m_{c1}\left(-\frac{\Delta C_{c1}}{\Delta E}\left(\omega \right)-\frac{\Delta C_{c2}}{\Delta E}\left(\omega \right)+\frac{\Delta C_{a}}{\Delta E}(\omega )\right)$$

(24) $$={-d}_{f}\left[\left(m_{c2}-m_{c1}\right)\frac{G_{c2}}{(\mathrm{j\omega}d_{f})+K_{c2}}+\left(m_{a}+m_{c1}\right)\frac{G_{a}}{(\mathrm{j\omega}d_{f})+K_{a}}+m_{s}\frac{G_{s}}{(\mathrm{j\omega}d_{f})+K_{s}}\right]$$

$${\left.\frac{\Delta m}{\Delta E}\right|}_{\mathrm{th}}^{c1c2s}\left(\omega \right)=\frac{∆m}{∆E}\left(\omega \right)-m_{a}\left(-\frac{\Delta C_{c1}}{\Delta E}\left(\omega \right)-\frac{\Delta C_{c2}}{\Delta E}\left(\omega \right)+\frac{\Delta C_{a}}{\Delta E}(\omega )\right)$$

(25) $$=-d_{f}\left[\left(m_{c1}+m_{a}\right)\frac{G_{c1}}{(\mathrm{j\omega}d_{f})+K_{c1}}+\left(m_{c2}+m_{a}\right)\frac{G_{c2}}{(\mathrm{j\omega}d_{f})+K_{c2}}+m_{s}\frac{G_{s}}{(\mathrm{j\omega}d_{f})+K_{s}}\right]$$

The transfer functions, $\frac{∆q}{∆E}\left(\omega \right)$, $\frac{∆E}{∆I}\left(\omega \right)$ and $\frac{∆m}{∆E}\left(\omega \right)$, obtained from ac-electrogravimetry are expressed in mC cm^−2^ V^−1^, kΩ cm^2^ and μg cm^−2^ V^−1^, respectively.

To illustrate typical experimental ac-electrogravimetry data derived from the $\frac{\Delta m}{\Delta E}\left(\omega \right)$ transfer function, a diagram is shown in Figure 13. For a single ion transfer at the interfacial region, a typical anion (loop 2) and cation (loop 1) appear on the III and I quadrants of the $\frac{\Delta m}{\Delta E}\left(\omega \right)$ transfer function, respectively (Figure 13a). In a more complex electrochemical process, the contribution of multiple species (loops 3–6) can occur (Figure 13b). In the latter case, different kinetic constants are also considered, e.g.*,* an anion faster than a cation (loop 4).

The transfer function $\frac{\Delta m}{\Delta E}\left(\omega \right)$ can also detect the contribution of free solvent ), which participates in charge compensation either (i) in the free form, following the same direction as the cation/anion flux, or (ii) as part of a solvation/hydration shell [68].

This technique has been widely used to study capacitive behavior in carbon materials. For instance, Escobar-Teran et al. [20] used ac-electrogravimetry to study the interfacial electrochemical processes of SWCNTs in detail (Figure 14), complementing conventional EQCM measurements (Figure 6). The experimental electrogravimetric transfer function, $\frac{\Delta m}{\Delta E}\left(\omega \right)$, fitted by the theoretical equations described previously, is presented in Figure 14A. Distinct species—including hydrated Na^+^, H_2_O, and H^+^—can be discriminated through changes in the frequency (Figure 14A). The ion-transfer kinetic constant, Ki, obtained from ac-electrogravimetric data, is also presented in Figure 14B.

The hydrated Na^+^ ions are surprisingly faster than protons (H^+^) (Figure 14B), suggesting that hydrated Na^+^ ions are more readily electrosorbed at the electrode/electrolyte interface. Furthermore, the total mass response obtained by addition of the individual contributions is shown in Figure 14C, which is in excellent agreement with the global EQCM results (Figure 14D). These findings indicate that ac-electrogravimetry provides a reliable gravimetric and dynamic deconvolution of the global EQCM responses.

Goubaa et al. [79] employed AC-electrogravimetry to conduct an in-depth investigation of capacitive charge storage mechanisms in electrochemically reduced graphene oxide (ERGO) electrodes, expanding upon preliminary EQCM observations (Figure 7). The authors demonstrated the critical role of solvation dynamics in electroadsorption kinetics, providing experimental evidence for the inverse relationship between ionic radius and hydration energy. Specifically, smaller cations with higher charge densities exhibit stronger ion–solvent interactions, requiring higher desolvation energies that ultimately hinder transfer kinetics. Consequently, Li^+^ ions (smaller ionic radius, higher hydration energy) undergo slower charge-transfer kinetics due to the energy barrier associated with partial or complete dehydration. In contrast, Na^+^ ions (larger ionic radius; lower hydration energy) require lower desolvation energy, which facilitates faster interfacial transfer kinetics across the electrode–electrolyte interface (Figure 15).

Escobar-Teran et al. [6] also used ac-electrogravimetry to study the effect of carbon nanotube structure (single- vs. multi-walled) on ion dynamics at the electrode/electrolyte interface (Figure 16). The carbon films were tested in NaCl aqueous electrolytes. Figure 16A,B show the experimental transfer functions $\frac{∆m}{∆E}\left(\omega \right)$ for DWCNT and MWCNT respectively, together with the corresponding theoretical fits based on equations previously reported in the literature [6,20,26]. As the frequency changes, different ionic species can be distinguished, as illustrated in Figure 16A,B. Concerning the transport kinetics, the results in Figure 16C,D demonstrate that hydrated Na^+^ ions undergo faster mass transfer than H^+^ ions in both types of carbon nanotubes. It is also observed that the transfer kinetics of free solvent (H_2_O) closely match those of chlorine ions at anodic potential.

In addition, the movement of hydrated Na^+^ and Cl^−^ ions is accompanied by the transport of free water molecules, a phenomenon that can be attributed to electrodragging [20,25]. Through ac-electrogravimetry, changes in the relative concentration of each species can be obtained (Figure 17), making it possible to distinguish the individual contributions of the different ionic species, as shown in Figure 17A,B. For MWCNTs, the relative concentration values of the free solvent (H_2_O) are higher than those of cationic and anionic species (Figure 17B), while in DWCNTs, the relative concentration values of the hydrated Na^+^ are higher than those of H_2_O and H^+^ at cathodic potentials (Figure 17B).

The findings demonstrated that DWCNTs are better candidates for charge storage capacity electrodes, as they can accommodate a higher concentration of charged species than MWCNTs.

Halim et al. [92] used ac-electrogravimetry to investigate the interfacial ion exchange of a composite electrode made of carbon nanotube/poly(ortho-phenylenediamine) (CNT/PoPD) in an aqueous NaCl electrolyte. The role of each species—including Na^+^ cations, Cl^−^ anions, and protons—participating in the charge compensation mechanism of CNT/PoPD was revealed with this technique. Furthermore, the findings highlighted the positive synergistic interaction between the CNT and the PoPD, revealing the creation of additional electro-active sites for ion exchange in the nanocomposite electrode.

Sel et al. [95] investigated the interfacial properties of 2D graphene–polydopamine (ERGO-PDA) composite electrodes in protic ionic liquid-based electrolytes ($\text{pyrrolidinium hydrogen sulfate},\left[{\mathrm{Pyr}}^{+}\right]\left[{\mathrm{HSO}}_{4}^{-}\right]$) using ac-electrogravimetry. The findings revealed that chaotropic Pyr^+^ ions hindered the approach of both kosmotropic Na^+^ ions and water molecules to the interface, thereby suppressing electrodragged water transport—a phenomenon prominently observed in pristine aqueous electrolytes. Furthermore, this electrogravimetric study experimentally demonstrated that mitigating water adsorption at the electrode/electrolyte interface enhances electrochemical energy storage performance in ERGO-PDA composite electrodes.

Recently, Su et al. [93] used ac-electrogravimetry to study the charge storage behavior of MWCNTs/PVDF, pure PEDOT films, and PEDOT/MWCNT composite films in an aqueous Na_2_SO_4_ solution. The study demonstrated that the synergy between MWCNTs and PEDOT improves charge storage compared with MWCNTs/PVDF-HFP and pure PEDOT films.

In summary, the ion dynamics in carbon materials during charge and discharge processes can be analyzed in detail using ac-electrogravimetry, providing valuable information about the mechanisms governing ion transport at the interfacial region.

### 2.4. Nuclear Magnetic Resonance Spectroscopy (NMR)

NMR spectroscopy is a powerful technique for studying the ion environments in supercapacitor electrodes and quantifying changes in the populations of adsorbed species during the charge–discharge cycles [96,97,98,99,100,101,102]. When ions are confined within nanopores, the NMR signal of the electrolyte ions can shift toward lower frequencies. This shift is associated with the presence of delocalized electrons on the carbon surface, which partially shield the nuclei from the applied magnetic field [5].

NMR data collected from supercapacitor electrodes can provide very useful information concerning ion behavior and charge–storage mechanisms. First, resonance intensities of in-pore ions can be used to estimate the populations of cations and anions, providing insights into pore composition and charge storage mechanisms (Figure 18, panel 1). Second, chemical shift differences between in-pore and ex-pore resonances can provide information concerning the structural characteristics of carbon material (Figure 18, panel 2). Third, the linewidth of the in-pore resonance can be related to the mobility and dynamics of ions confined within the pore (Figure 18, panel 3) [96].

Forse et al. [101] used NMR spectroscopy to investigate ion dynamics and charge storage in supercapacitors containing ionic liquid electrolytes. Their results indicated that anion adsorption and desorption play a more significant role in the charge–storage mechanism than the corresponding processes involving cations. Later, Forse et al. [97] investigated the ion dynamics in supercapacitor electrodes using in situ diffusion NMR spectroscopy. Their findings showed that both electrolyte concentration and carbon pore-size distribution influence ion diffusion inside the pores, as well as the exchange of ions between the nanopores and the surrounding electrolyte. These results contribute to a better understanding of the relationship between the structural properties of electrode materials, ion transport, and the overall performance of supercapacitors.

Combining in situ NMR and EQCM was also employed to study the charge storage mechanism of microporous carbon YP50F in tetraethylphosphonium tetrafluoroborate with acetonitrile in [22]. Two charging mechanisms were identified depending on the electrode polarity. During negative polarization, charge was stored via counter-ion (cation) adsorption, whereas ion exchange dominated during positive polarization. Consistently, EQCM measurements suggested concomitant solvent reorganization within the pores without a net solvent flux in or out of the porous network. This combined approach provided direct, molecular-level insights into the charging process in carbon micropores.

Balhatchet et al. [103] used ex situ NMR and operando EQCM experiments to investigate the charge storage mechanism in Ni_3_(HITP)_2_MOF supercapacitors, revealing that cations are the main contributors to charge storage, while anions contribute to a lesser extent.

Huang et al. [104] used in situ EQCM and NMR to elucidate the co-intercalation process and electrochemical behavior of solvated Na^+^-diglyme ([Na-*x*G2]^+^) in graphite. The combination of these techniques provided key molecular-level insights into the spatial evolution of solvated ions within the graphite layers during co-intercalation. The authors concluded that quantitatively monitoring both mass changes and ionic species is crucial for understanding structure–performance relationships and desolvation dynamics at the interfacial region.

### 2.5. Computer Simulations

The simulation of electrochemical systems has become an increasingly useful tool for gaining a deeper understanding of the molecular processes involved in energy-storage devices [105,106]. These simulations can be used to investigate both bulk electrode materials and electrolytes, helping to characterize their properties and understand their behavior. More recently, increasing attention has been given to using computational approaches to better describe and explain the mechanisms responsible for charge storage [105].

The most widely used classical framework to describe charge storage mechanisms is the Gouy–Chapman–Stern model [107,108]. This model integrates two earlier theoretical descriptions: the Helmholtz and the Gouy–Chapman model (Figure 19). In the Helmholtz model, the electrical double layer (EDL) is treated as a single layer of counter-ions located a certain distance from the electrode. Within this layer, the potential varies linearly, and the corresponding capacitance, *C_H_*, is directly proportional to the electrode surface area and inversely proportional to the separation distance between the electrode and the Helmholtz layer [109].

In contrast, the Gouy–Chapman model accounts for the diffusion of ions within the solvent. In this case, the potential decays exponentially from the electrode surface, producing a capacitance *C_GC_* that depends on the electrode potential at the Stern plane and the bulk ion concentration [110,111].

The overall capacitance of the EDL is obtained by treating these two capacitances as capacitors connected in series. When determining the total capacitance of an electric double-layer capacitor, the capacitances of the two EDLs formed at each electrode must also be considered in series, as expressed in the following equation [112]:

(26) $$\frac{1}{C}=\frac{1}{C_{H}}+\frac{1}{C_{\mathrm{GC}}}$$

where *C_H_* is the inner layer or Helmholtz capacitance, while *C_GC_* corresponds to the diffuse layer capacitance, also known as Gouy–Chapman capacitance.

Despite its usefulness, the Stern-based description has several limitations. For example, it does not adequately account for the influence of ion concentration on capacitance or for the specific adsorption of ions on the electrode surface [113,114]. Furthermore, it is mainly applicable to equilibrium conditions and does not provide insight into the dynamic behavior of electrolyte species.

Computational modeling methods such as molecular dynamics (MD) [115,116], Monte Carlo (MC) simulations [113], and density functional theory (DFT) [117] are being used to understand the charging and discharging of supercapacitors. In particular, MD simulations can provide a better description of the EDL and also calculate the movement of particles. It has been reported that combining theoretical calculations with in situ experimental measurements provides a deeper understanding of how charging occurs [27].

Merlet and co-workers used molecular dynamics (MD) simulations to investigate the different morphological regions found in carbide-derived carbon (CDC) materials, including edge, plane, hollow, and pocket sites, and to evaluate how strongly ions are confined within these structures [118]. Anion confinement inside narrow pores fundamentally changes desolvation mechanisms compared to bulk behavior. Applied potential further amplifies electrowetting effects on light in the smallest nanopores. Together, these observations establish a clearer picture of ion confinement by showing how carbon pore geometry governs ionic spatial distribution and transport rates, offering a framework to inform future theoretical and empirical studies.

To properly describe the porous structure of carbon-based materials, density functional theory (DFT) is widely used to estimate pore-size distributions and accessible surface areas from gas adsorption measurements [119,120,121]. For carbon materials with irregular surfaces and a high concentration of functional groups, quenched solid density functional theory (QSDFT) can provide a more accurate description of both micropores and mesopores [122,123,124]. In addition, classical DFT and molecular DFT (MDFT) are useful for estimating the free energy of solvents, which is particularly important for understanding ion desolvation when electrolytes are confined within nanopores [125,126,127].

Many computational methods have been used to model ion behavior when confined to porous electrodes. Among these approaches, Monte Carlo (MC) simulations use statistical sampling to map energetically favorable ion configurations, balancing charge density, potential energy and dynamic pairing. By incorporating ion–electrode interaction forces, MC modeling provides a realistic picture of ionic behavior ranging from simple migration and electrolyte exchange to electrode insertion and charge redistribution across opposing electrodes. However, millions of simulation steps are required before the system reaches equilibrium [128,129,130,131].

MC simulations have been applied to different electrode structures, ranging from flat surfaces to highly porous materials, and to a variety of electrolytes. They have contributed significantly to the understanding of the superionic state of ionic liquids and have also revealed oscillations in capacitance [132]. Later, Kornyshev’s group used MC simulations to investigate how ionophilic and ionophobic slit-shaped nanopores affect the energy density of supercapacitors [130]. A major advantage of Monte Carlo simulation over standard DFT is its ability to map complex 3D carbon networks. This gives a much clearer, more realistic view of what actually happens at the electrode–electrolyte boundary [133].

Molecular dynamics (MD) simulations provide a powerful lens for probing electric double-layer capacitors (EDLCs) at the atomic scale. By propagating ion trajectories through Newton’s laws of motion, these simulations reveal how the electrolyte responds right at the electrode boundary [134]. Two main approaches have been used to represent polarized electrodes. In early MD studies, the carbon atoms were generally assigned fixed charges [135,136]. However, in 2012, Salanne’s group showed that the behavior of the electrolyte can be significantly different depending on whether the electrode is modeled under constant-charge or constant-potential conditions. In particular, the constant-potential approach provides a more realistic representation of porous carbon electrodes and their interaction with the electrolyte [137].

Following the discovery of unusually high capacitance in subnanometer pores, MD simulations became particularly useful for understanding how electrolytes are organized within carbon nanopores. These studies revealed the molecular structure of ionic liquids confined in disordered microporous carbons and later examined ion configurations in both micro- and mesopores using different ionic liquid systems [138,139].

One of the main strengths of MD simulations is their ability to reproduce and analyze the charging dynamics inside nanopores. Three fundamental processes have been identified: counter-ion adsorption, co-ion desorption, and ion exchange. MD simulations have established that ion transport and dynamics are crucial for determining supercapacitor performance. Consequently, the high capacitance observed in nanopores and their fast-charging power capabilities require optimizing both the structural features of the carbon material and the charge storage mechanisms [140,141,142]. In addition, the rate at which ions diffuse through the pores is closely related to the power performance of the supercapacitor [143,144]. Interestingly, Kondrat and co-workers proposed that subnanometer ionophobic pores could promote faster charging [143]. This idea was later supported by additional computational studies, providing further evidence of the important role of pore structure in ion transport and charge–storage performance. This challenges the conventional assumption that larger pores necessarily enable faster charging and opens new avenues for designing ionophobic porous materials that combine high energy and high-power densities [145].

Despite their strengths, classical coarse-grained MC and MD approaches often simplify ions as spherical particles to reduce computational cost. For more detailed insights into ion–carbon interactions at polarized interfaces, especially during charging, atomistic ab initio methods are required. These computational approaches take into account important characteristics such as molecular geometry, polarization effects, and the electronic structure of carbon-based materials. When the electrode is polarized, changes in its electronic structure and in the electrostatic interactions with the electrolyte can modify the screening potential and, consequently, the structure of the electric double layer. Density functional theory (DFT) and other advanced DFT-based approaches are commonly used for these types of ab initio calculations. In addition to providing information about the structure of porous carbons, DFT has helped explain why capacitance can increase in nanopores, particularly due to the effects associated with positive surface curvature [146,147]. More recently, DFT studies have also provided valuable insights into the structure of electric double layers and the behavior of electrode/electrolyte interfaces [148].

Mesoscopic lattice models have also recently emerged as useful tools for describing the structural, dynamic, and capacitive behavior of EDLC systems [142]. Their main advantage is their high computational efficiency, as they can run approximately 10,000 times faster than conventional molecular simulations while combining information obtained from both simulations and experiments. These models have been successfully applied to examine how factors such as solvation and pore size affect ion adsorption, capacitance, and diffusion in both neat and solvated ionic liquid electrolytes. As these models continue to be improved, they could become increasingly valuable for studying the complex behavior of electrochemical systems.

At the same time, artificial neural networks and machine learning approaches are gaining attention as promising tools for predicting the capacitance of carbon-based electrodes [149,150]. It has been reported that combining experimental results with key parameters like specific surface area, pore-size distribution, and operating voltage can help predict electrode performance. This makes it more useful to compare different carbon materials and identify the properties that are most suitable for supercapacitor applications.

It has also been reported that combining DFT and ML offers a powerful approach to accelerating the discovery of new materials and improving the overall performance of supercapacitors. Expanding this scope to include molecular dynamics and Monte Carlo simulations strengthens both analytical depth and predictive power. Combining these multi-scale computational tools with machine learning allows researchers to fully capture complex electrode behavior while dramatically speeding up candidate material discovery [151].

## 3. Critical Analysis

The characterization techniques discussed above play a crucial role in elucidating interfacial charge storage mechanisms. However, despite their considerable potential, their implementation presents distinct strengths and limitations that require detailed analysis. A comparative summary of these techniques is presented in Table 1.

## 4. General Discussion

Unraveling interfacial charge storage mechanisms via in situ techniques is critical for advancing supercapacitor design. Integrating computational modeling with experimental findings offers a transformative approach to developing high-performance electrode materials. In this context, we analyze the different techniques described in the previous sections, particularly their strengths and limitations, and propose strategic combinations to better understand these charge storage mechanisms.

**(i)**  **Electrochemical impedance spectroscopy (EIS)**

Probing frequency-dependent interfacial responses makes EIS indispensable for isolating solution resistance, charge-transfer kinetics, and porous-electrode capacitance [152]. However, assigning circuit elements to distinct physical phenomena in porous carbons remains tricky. Distributed ion transport within complex networks, for instance, often mimics Warburg-like behavior without involving true semi-infinite diffusion [153,154]. While transmission-line and porous-electrode models offer far better physical realism than basic circuits, equivalent-circuit fitting is inherently non-unique—several distinct networks can yield identical spectra [152,155]. Extracting reliable mechanisms thus demands physically grounded models rather than high statistical fit parameters [153].

**(ii)**  **EQCM and ac-electrogravimetry**

By linking charge to mass changes via the Sauerbrey relation, EQCM pinpoints whether charging driven by adsorption, insertion, expulsion, or solvent flux dominates [65,156,157]. AC-electrogravimetry expands this capability by resolving transfer functions to extract kinetic coefficients for individual species [158]. That said, Sauerbrey calculations rely on rigid, uniform films. Swelling, viscoelastic shifts, and solvent coupling routinely skew raw mass values. Tracking dissipation alongside frequency (EQCM-D) resolves this by separating real mass changes from structural viscoelastic shifts [159].

**(iii)**  **In situ NMR spectroscopy**

NMR resolves molecular environments that pure electrochemistry misses, directly tracking chemical shifts and diffusion to reveal ion coordination, confinement, and solvation state within nanopores [160,161]. These structural insights are invaluable, yet spectral changes must be correlated with electrochemical curves rather than treated as isolated proofs of a mechanism [162,163].

**(iv)**  **Molecular Dynamics (MD) and Density Functional Theory (DFT)**

MD captures ion dynamic distribution, mobility, and pore desolvation, while DFT details adsorption energies and surface interactions [164,165,166]. Together, they clarify performance differences between electrolytes [167]. Still, atomistic models rely on assumed force fields and simplified pore geometries that overlook the structural disorder of real carbons [164,168]. Simulations excel at generating hypotheses, but they require experimental validation [169].

In summary, no single technique captures the complete physical picture of interfacial charge storage. Standard EQCM measures net mass changes but cannot resolve simultaneous counter-ion and co-ion exchange. On the other hand, in situ NMR spectroscopy quantifies ion populations inside micropores, though it lacks the time resolution needed for fast interfacial kinetics. While molecular simulations bridge these empirical gaps, they require experimental parameters for boundary validation.

To address these limitations, we highlight two distinct multi-scale approaches:

- **Route 1 (Electrogravimetric–Mechanical–Simulation Workflow):** A dynamic approach combining ac-electrogravimetry, EQCM-D, and constant-potential MD/DFT modeling. Here, EQCM-D monitors viscoelastic changes to verify film rigidity, ensuring the validity of Sauerbrey assumptions. Simultaneously, ac-electrogravimetry separates ionic species in the frequency domain, accounts for uncharged solvent transport, and calculates rate constants (*K_i_*). These experimental values are then fed into MD/DFT models to build a realistic atomistic representation of the electrical double layer.
- **Route 2 (Spectroscopic–Simulation Workflow):** A structural framework combining in situ/operando NMR spectroscopy with MD/DFT calculations. This established route uses chemical shifts and line broadening to measure ion confinement and equilibrium populations in porous networks, supported by calculated density profiles.

## 5. Summary and Future Outlook

Elucidating charge storage mechanisms in supercapacitors requires merging multi-scale in situ diagnostic tools with computational modeling to map the ion dynamics at the electrode/electrolyte interface.

**Mechanisms and diagnostic techniques**

- **Main charge–storage mechanisms:** Supercapacitors store energy mainly through two mechanisms. In EDLCs, charge is stored electrostatically through the reversible accumulation of ions at the electrode/electrolyte interface, without Faradaic reactions. In pseudocapacitors, storage involves rapid and reversible Faradaic redox reactions and ion intercalation within the electrode material.
- **Ac-electrogravimetry (EIS + QCM):** By coupling electrochemical impedance spectroscopy (EIS) with quartz crystal microbalance (QCM) measurements in the frequency domain, ac-electrogravimetry allows changes in mass to be correlated with the applied potential. One of its main advantages is that it can distinguish the contributions of individual ionic species from those associated with neutral solvent molecules. It also provides information about species-specific kinetic parameters and transfer resistances, which are difficult to obtain using conventional steady-state EQCM measurements.
- **Complementary in situ techniques:** In situ NMR spectroscopy can distinguish ions confined within nanopores from those present in the bulk electrolyte through changes in their chemical shifts. This approach provides useful insights into how ions are distributed, organized, and transported within the electrode during the charging process.
- **Computational Modeling (Constant-Potential MD and DFT):** This probes ion behavior under sub-nanometer confinement, directly resolving key interfacial phenomena such as partial desolvation, in-pore electrolyte restructuring, and electric field screening within complex pore architectures.

**Future Challenges and Outlook**

- **Improving atomistic and polarizability models:** Future studies should move beyond simplified spherical ion models by incorporating more advanced approaches, such as ab initio MD and DFT. These methods can better account for molecular geometry, solvent polarizability, and changes in the electronic structure of porous carbon electrodes under applied potential.
- **Understanding multistep interfacial processes:** A more detailed description of ion-transfer mechanisms is needed, particularly to distinguish processes such as cation/anion exchange from simultaneous co-adsorption. Bridging experimental electrogravimetry (ac-electrogravimetry, EQCM-D) or NMR spectroscopy with MD/DFT simulations yields a robust multi-scale approach. By combining real-time tracking of mass changes, ion kinetics, and viscoelastic behavior with molecular-level ion counting, this hybrid framework fully maps the dynamic processes governing the interfacial region.
- **Predictive machine learning:** Integrating machine learning with mesoscopic network modeling creates a practical pipeline for targeted electrode design. Rather than relying on trial-and-error, this connection directly maps how complex pore architectures and electrolyte chemistry dictate overall device performance—a key step toward accelerating the discovery of custom carbon materials.

## Figures and Tables

**Figure 1:**
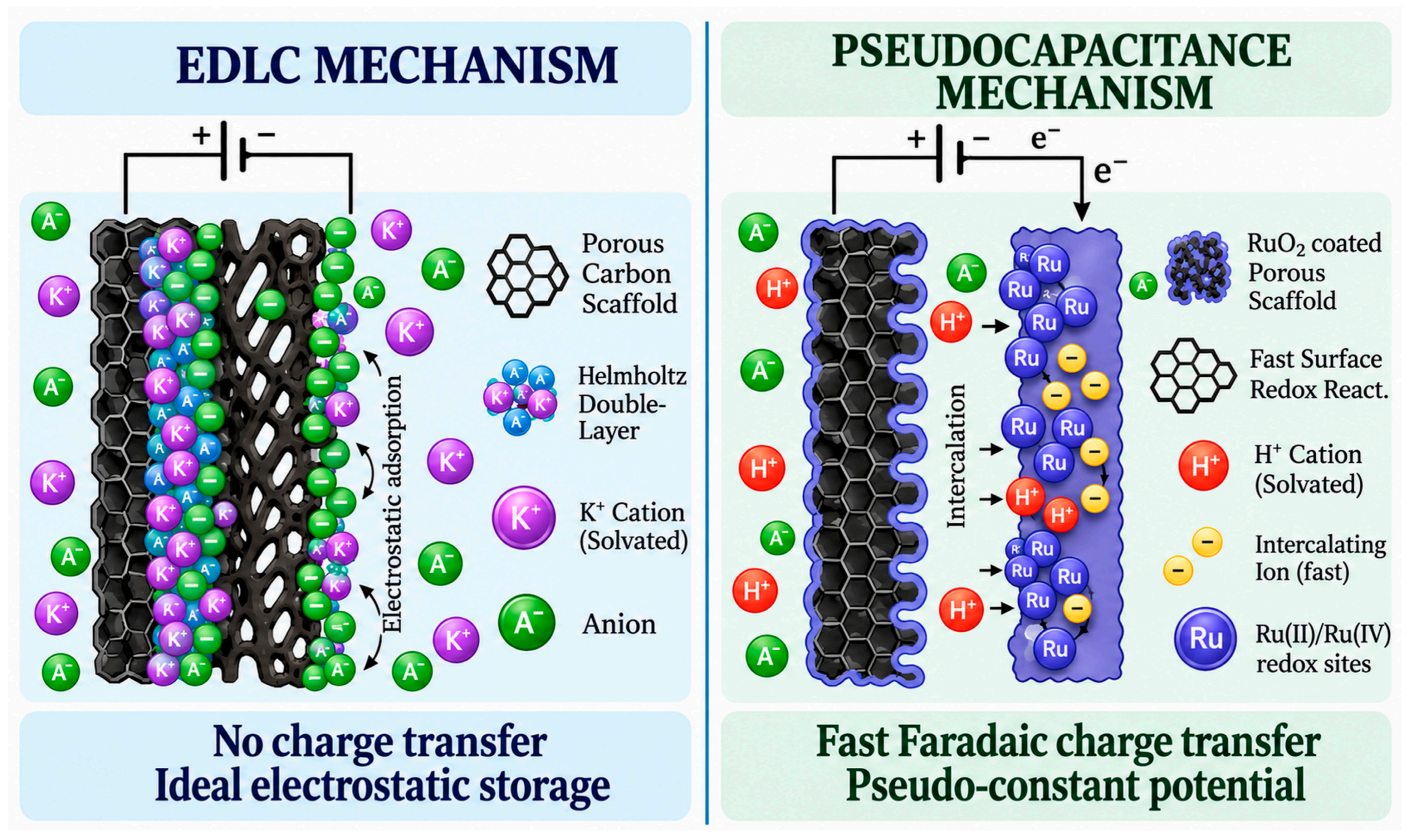
Principle of operation of an EDLC (**left**) and pseudocapacitor (**right**). Black curved and straight arrows denote electrostatic adsorption dynamics, electron transfer (*e*^−^), and fast ion intercalation pathways.

**Figure 2:**
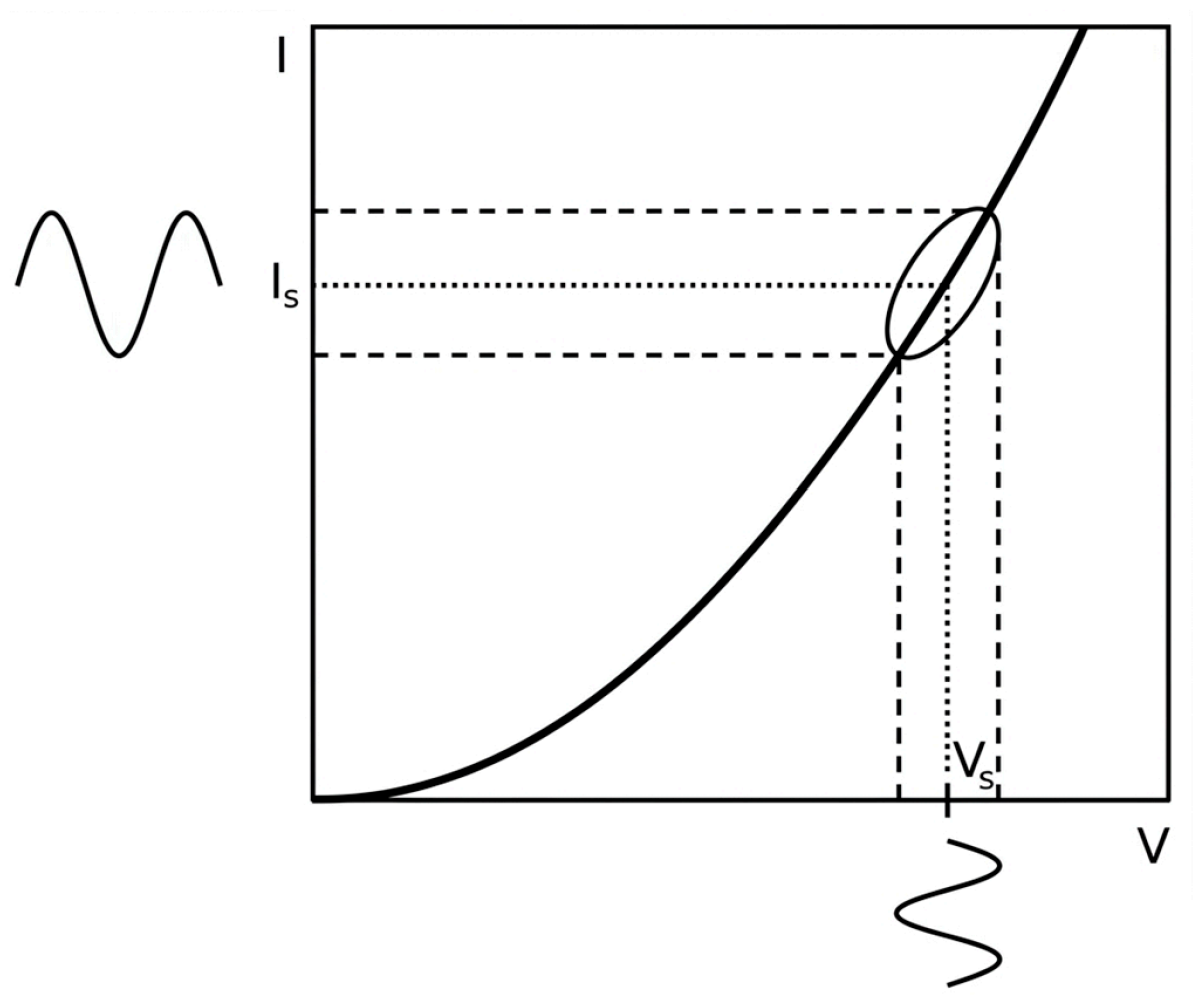
Linear current response to a low-amplitude sinusoidal perturbation potential around a stationary value. Adapted with permission from Ref. [47]. 2016 Escobar-Teran, F.D., PhD thesis, Pierre and Marie Curie University—Paris VI.

**Figure 3:**
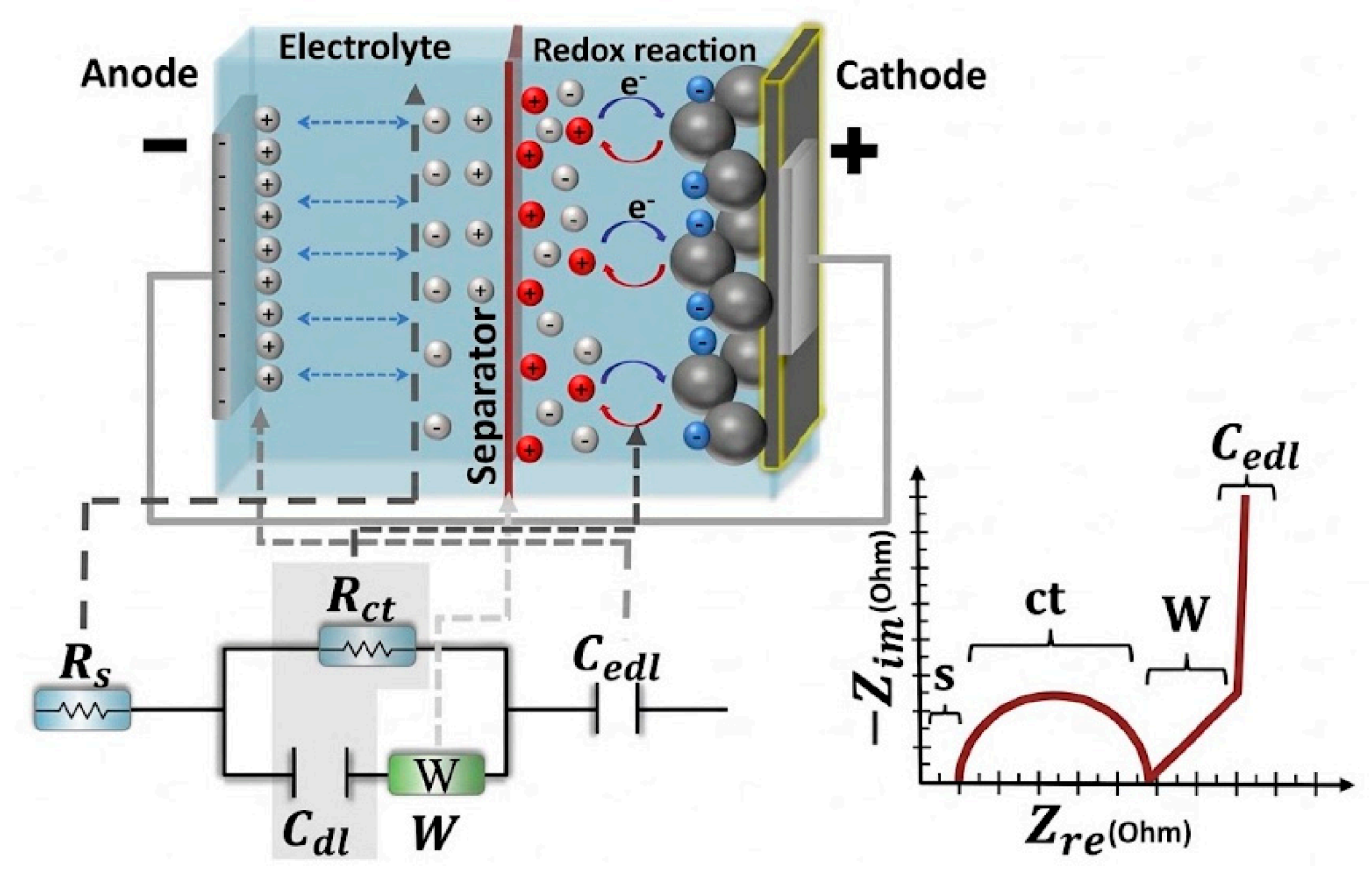
Equivalent circuit model and corresponding Nyquist plot regions for a hybrid supercapacitor, illustrating the electrolyte resistance (*R_s_*), double-layer capacitance (*C_dl_*), charge-transfer resistance (*R_ct_*), Warburg diffusion impedance (*W*), and low-frequency capacitive storage (*C_edl_*). The schematic shows the Anode (−), Cathode (+), Separator, ion diffusion (blue arrows), and redox reactions (*e*^−^). Dashed gray lines link cell regions to circuit components. In the Nyquist plot, *s* is the series resistance, *ct* the charge-transfer semicircle, *W* the Warburg region, and *C_edl_* the capacitive slope. Adapted with permission from Ref. [51] (copyright 2024) Royal Society of Chemistry.

**Figure 4:**
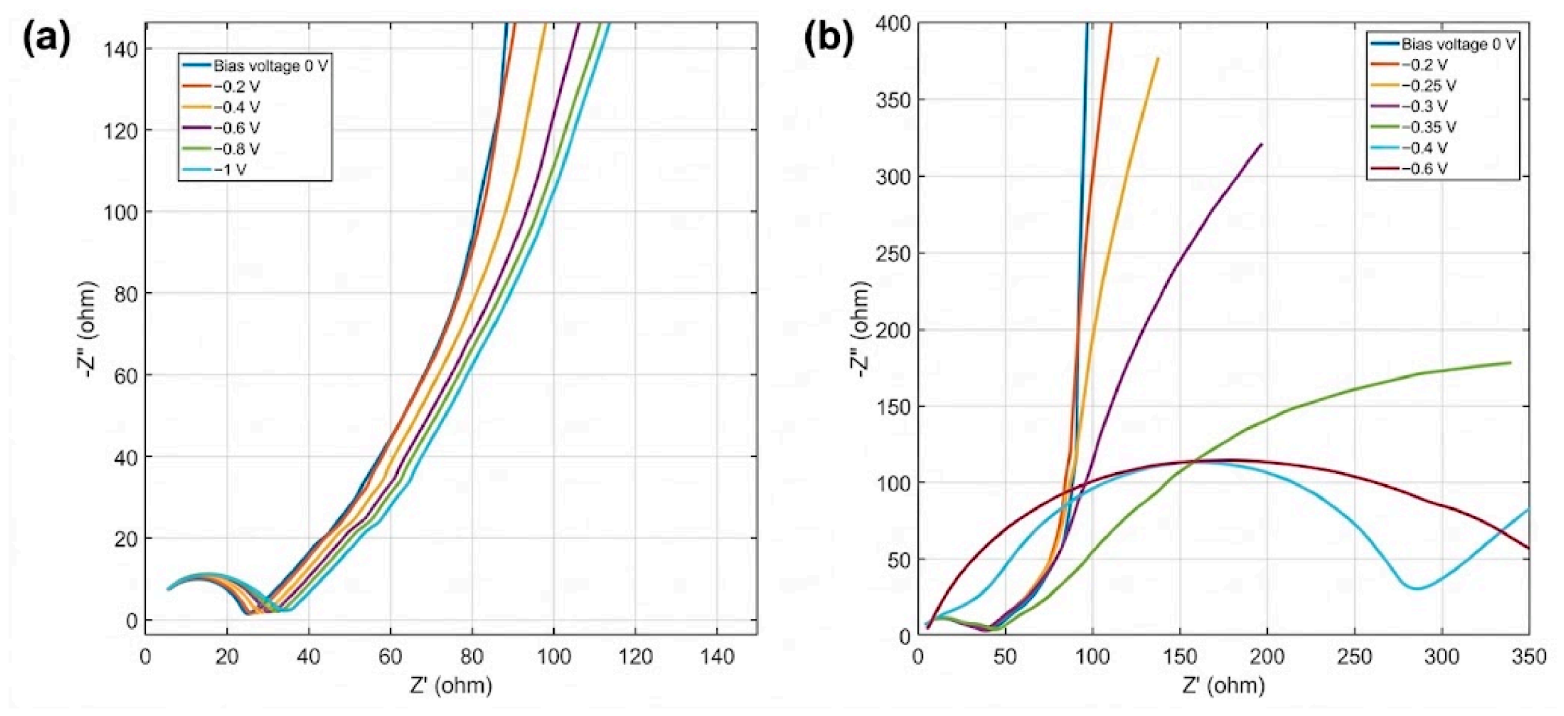
Nyquist plots of the MXene electrode derived from EIS measurements at different applied bias potentials in the (**a**) cathodic and (**b**) anodic potential ranges [60]; open access.

**Figure 5:**
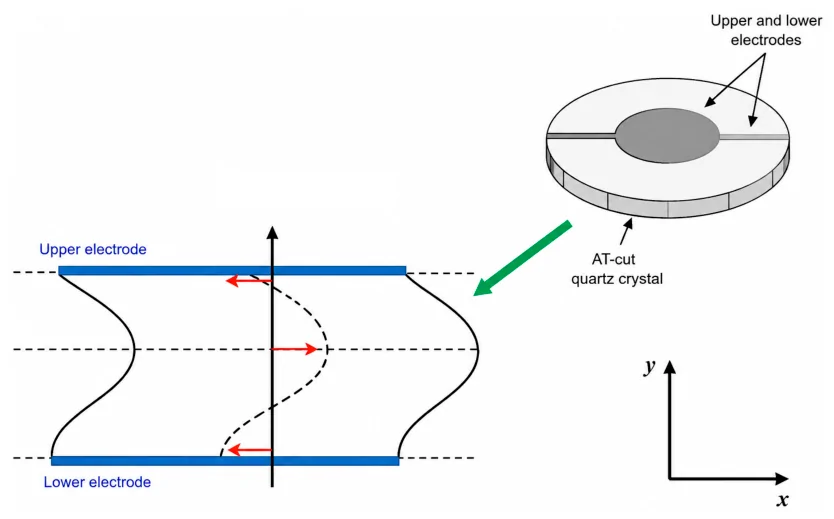
Schematic illustration of a thickness shear mode quartz resonator showing the resonant frequency. The upper diagram shows the quartz crystal disk with the upper (dark gray) and lower (light gray) metallic electrodes. The green arrow links the physical sensor to the cross-sectional view of the acoustic wave propagation along the *y*-axis. Blue solid lines represent the upper and lower electrodes bounding the crystal; horizontal dashed lines indicate the nodal and interface planes. The curved solid and dashed lines depict the fundamental shear deformation wave pattern across the crystal thickness, while red horizontal arrows denote the direction and magnitude of particle displacement. Adapted from Escobar-Teran, F.D., PhD thesis, Pierre and Marie Curie University—Paris VI, 2016 [47].

**Figure 6:**
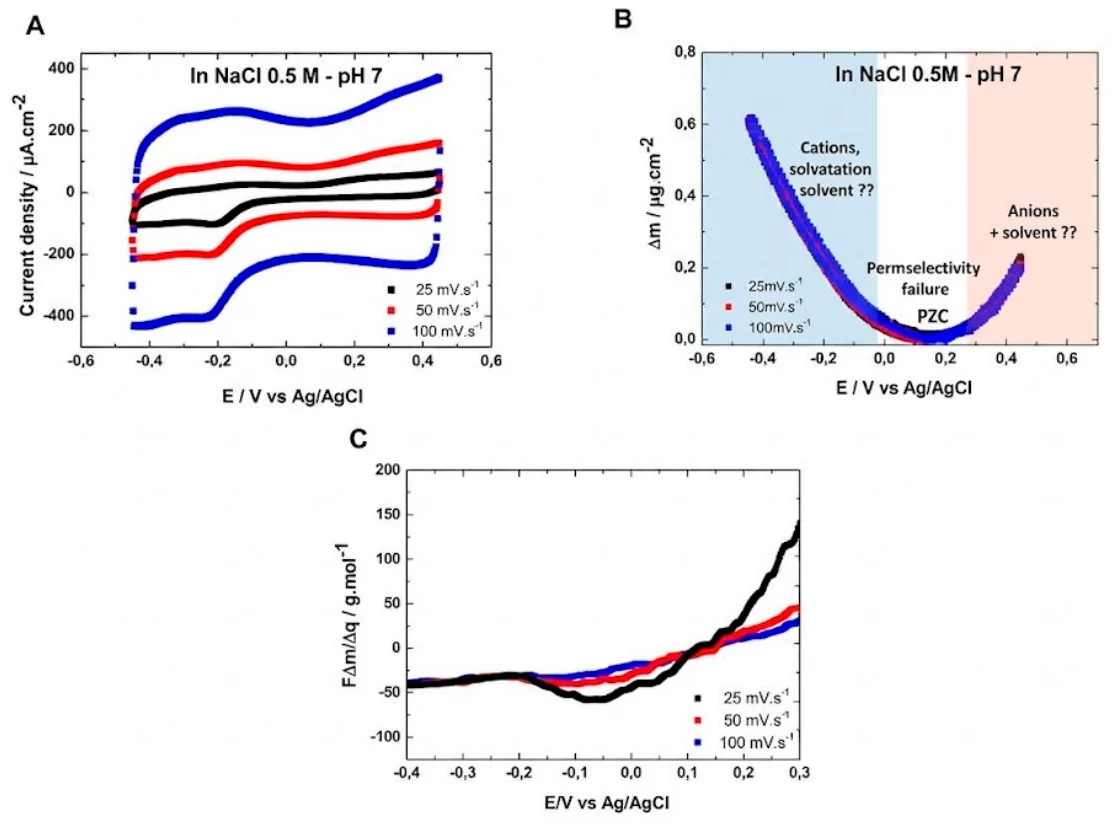
CV curves (**A**), simultaneous mass responses (**B**), and $F\times \frac{∆m}{∆q}$ function (**C**) of SWCNT-based thin films in 0.5 M NaCl. Reproduced from Ref. [20] with permission from Elsevier (copyright 2016).

**Figure 7:**
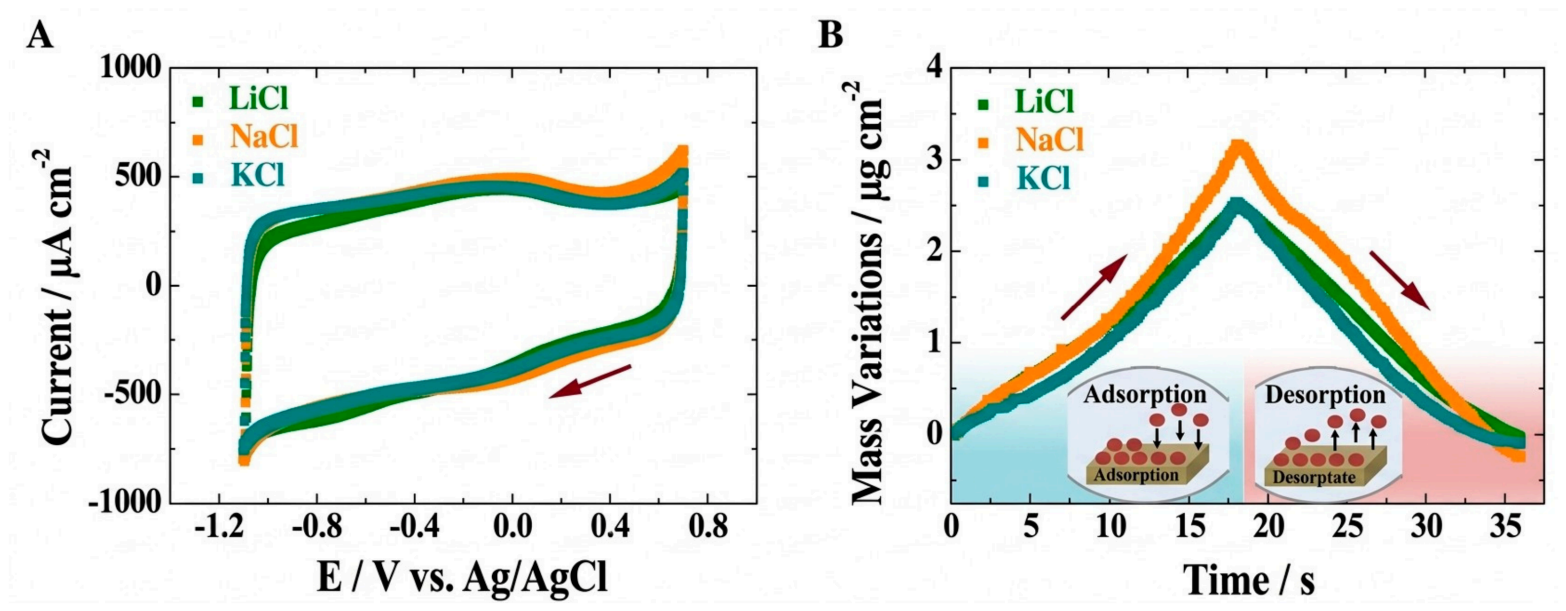
(**A**) Cyclic voltammetry (CV) profiles and (**B**) corresponding real-time mass variations in thin ERGO films recorded in 0.5 M aqueous LiCl, NaCl, and KCl solutions at a scan rate of 100 mV s^−1^. Arrows in panel (**A**) denote the potential sweep direction, starting from anodic to cathodic values. Adapted from Ref. [79] with permission of the American Chemical Society (copyright 2017).

**Figure 8:**
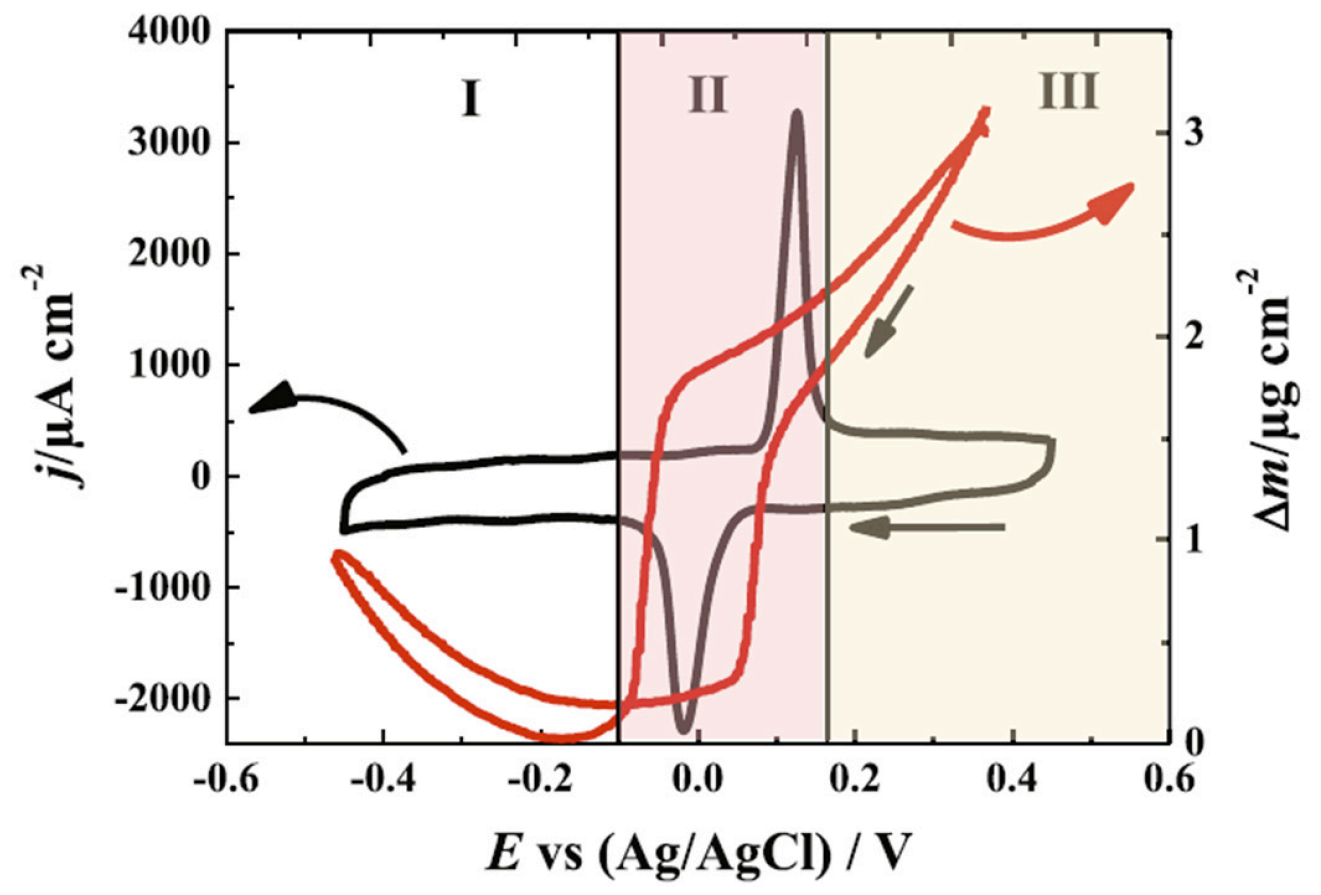
The EQCM data for SWCNT/PB nanocomposite films include current–potential and mass–potential profiles recorded in a 0.5 M KCl aqueous electrolyte at pH 3, using a scan rate of 50 mV s^−1^. The black line corresponds to the current density (left axis) and the red line to the mass change (right axis), with arrows indicating the corresponding y-axes and scan directions. Roman numerals (I, II, and III) denote different potential zones associated with distinct electrochemical regimes. Reproduced from Ref. [24] with permission from Elsevier (copyright 2018).

**Figure 9:**
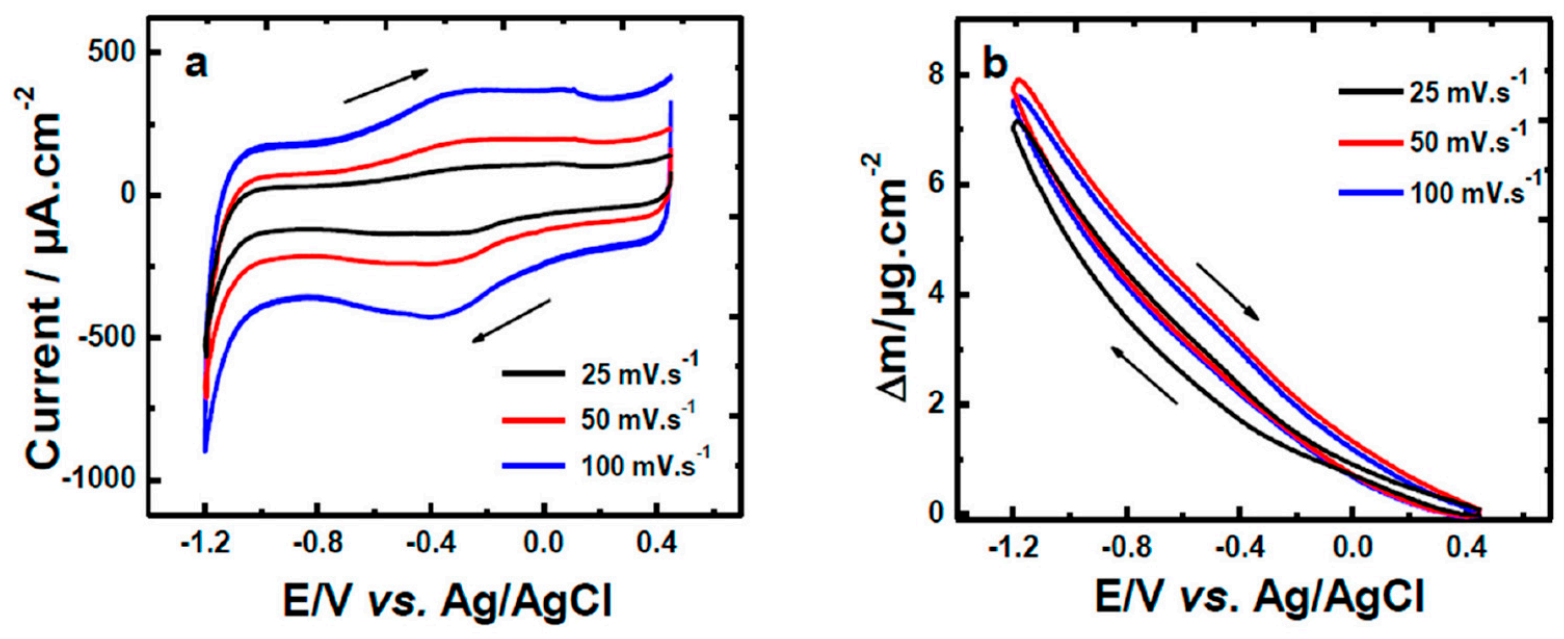
Electrogravimetric response of SWCNT/PPy composite thin films in 0.5 M aqueous NaCl: (**a**) cyclic voltammograms and (**b**) corresponding mass changes as a function of potential. Arrows in panel (**a**) represent the sweep direction, initiated from anodic to cathodic potentials [26]; open access.

**Figure 10:**
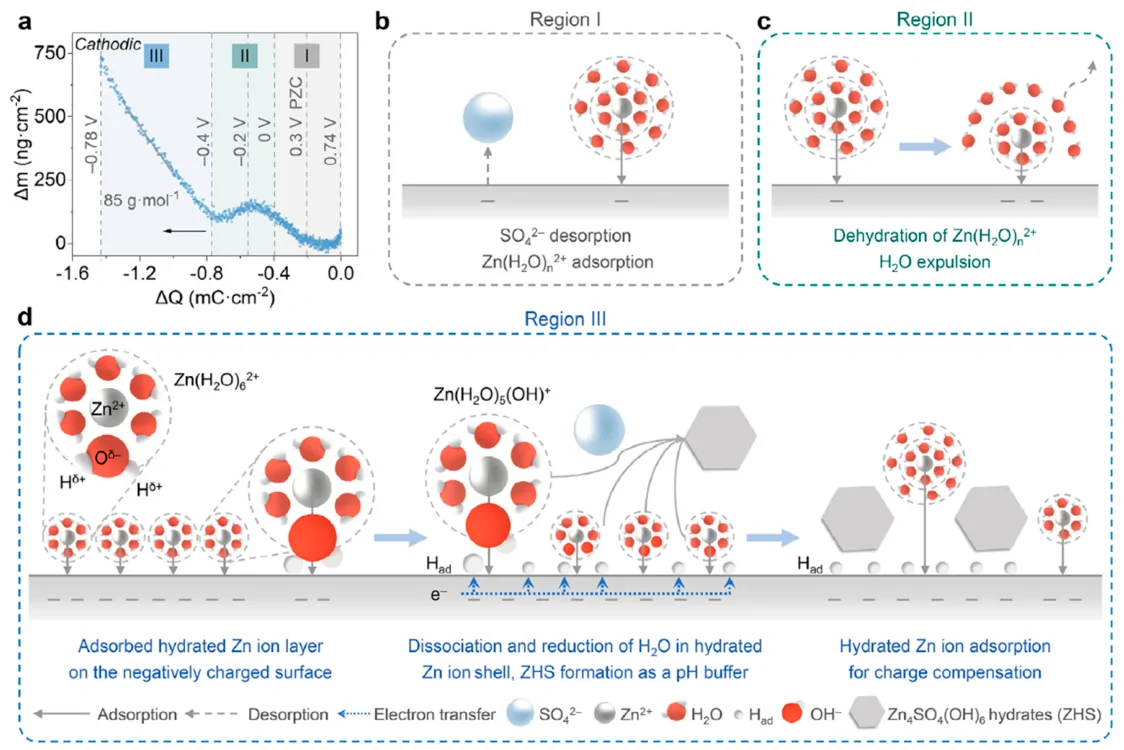
Multistep charge storage behavior in porous carbon. (**a**) EQCM analysis of UC1:5 deposited on a gold-coated quartz crystal acting as the working electrode in a 2 M ZnSO_4_ aqueous electrolyte, with Zn foil serving as the counter electrode and a saturated calomel electrode (SCE) as the reference. The variation in electrode mass (Δm) as a function of accumulated charge (ΔQ) is shown during the cathodic sweep of cyclic voltammetry. The arrow indicates the direction of polarization, and three distinct regions are highlighted with different colors. Vertical dashed gray lines denote the potential limits separating each region. (**b**–**d**) Schematic representation of the charge storage mechanisms occurring across the different potential ranges (Regions I, II, and III) [83]; open access.

**Figure 11:**
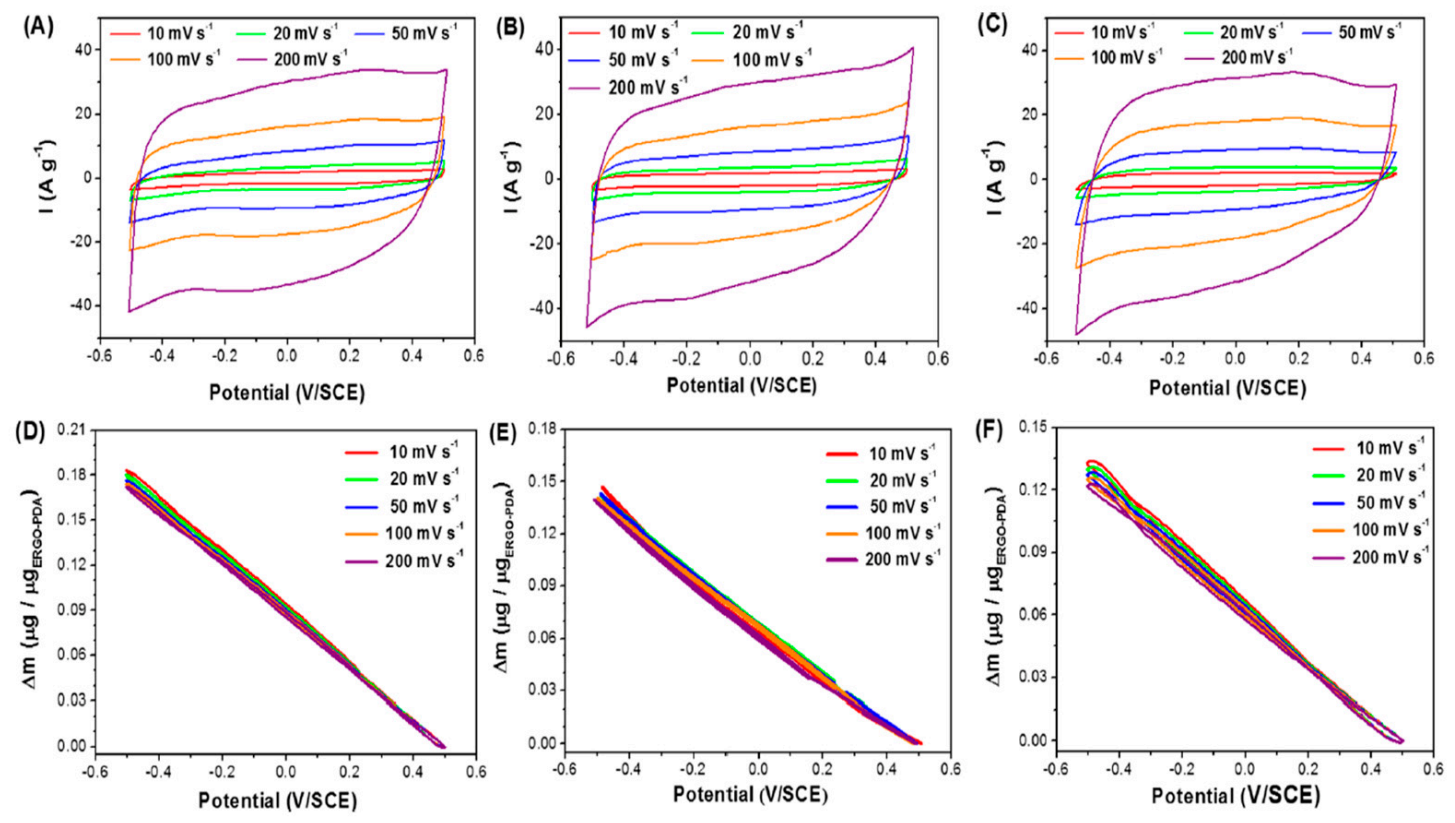
(**A**–**C**) Cyclic voltammetry profiles and (**D**–**F**) mass variation in the ERGO–PDA electrode as a function of potential in a 0.5 M C_2_SO_4_ aqueous electrolyte, where C corresponds to (**A**,**D**) K, (**B**,**E**) Na, and (**C**,**F**) Li [84]; open access.

**Figure 12:**
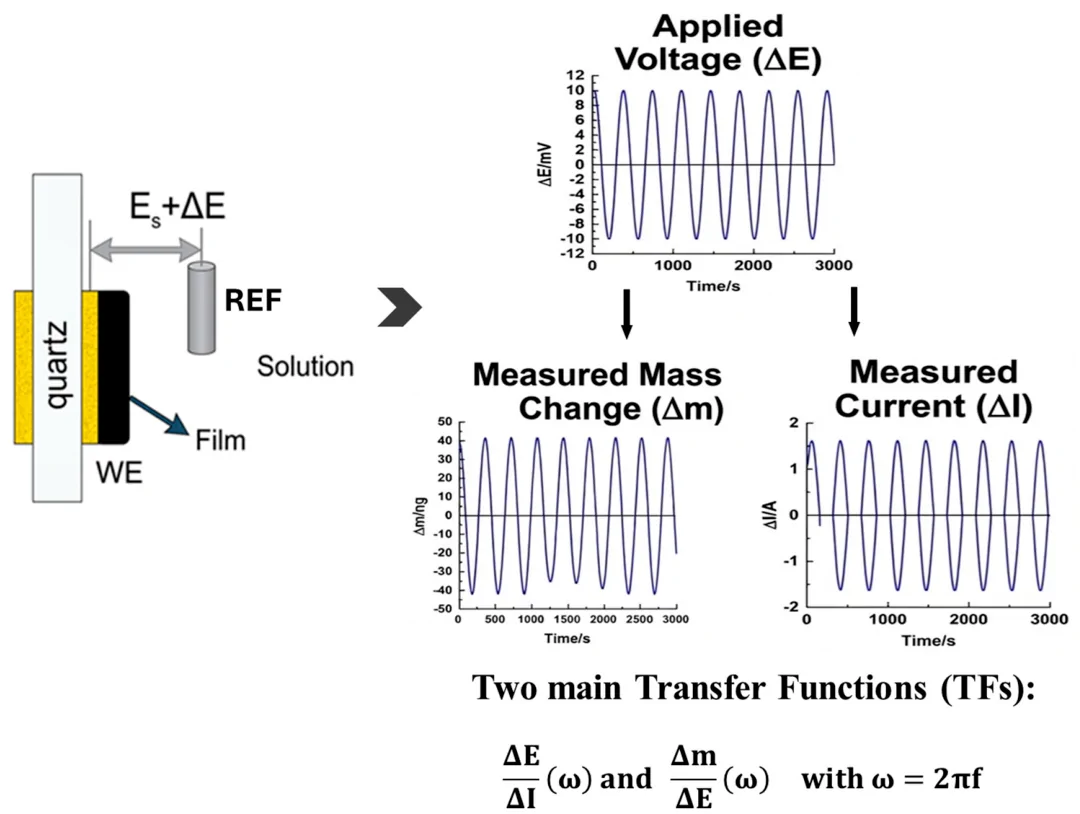
Principle of ac-electrogravimetry. The arrows denote the applied potential perturbation and the resulting measured mass and current responses, leading to the main experimental transfer functions. Adapted from Escobar-Teran, F.D., PhD thesis, Pierre and Marie Curie University—Paris VI, 2016 [47].

**Figure 13:**
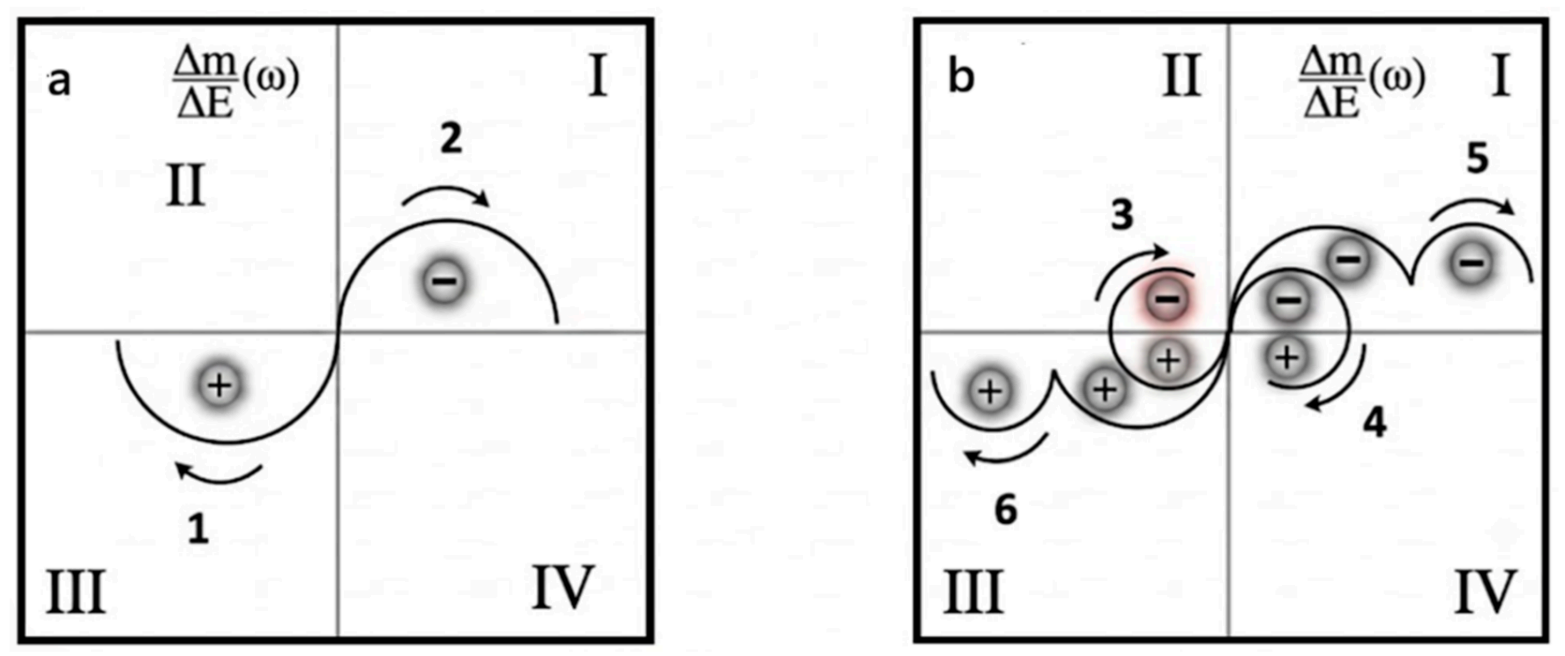
Schematic representation of the experimental transfer function for $\frac{\Delta m}{\Delta E}\left(\omega \right)$. The response of one cation and one anion is indicated in (**a**). The response of multiple species, including different kinetic constants, is indicated in (**b**). Roman numerals (I–IV) denote the four quadrants of the complex plane. Circled plus (+) and minus (−) symbols represent cationic and anionic species contributions, respectively. Numbered arrows (1–6) indicate the direction of increasing angular frequency (*ω*) for each process. Adapted from Gao, W., PhD thesis, Sorbonne University, 2018 [94].

**Figure 14:**
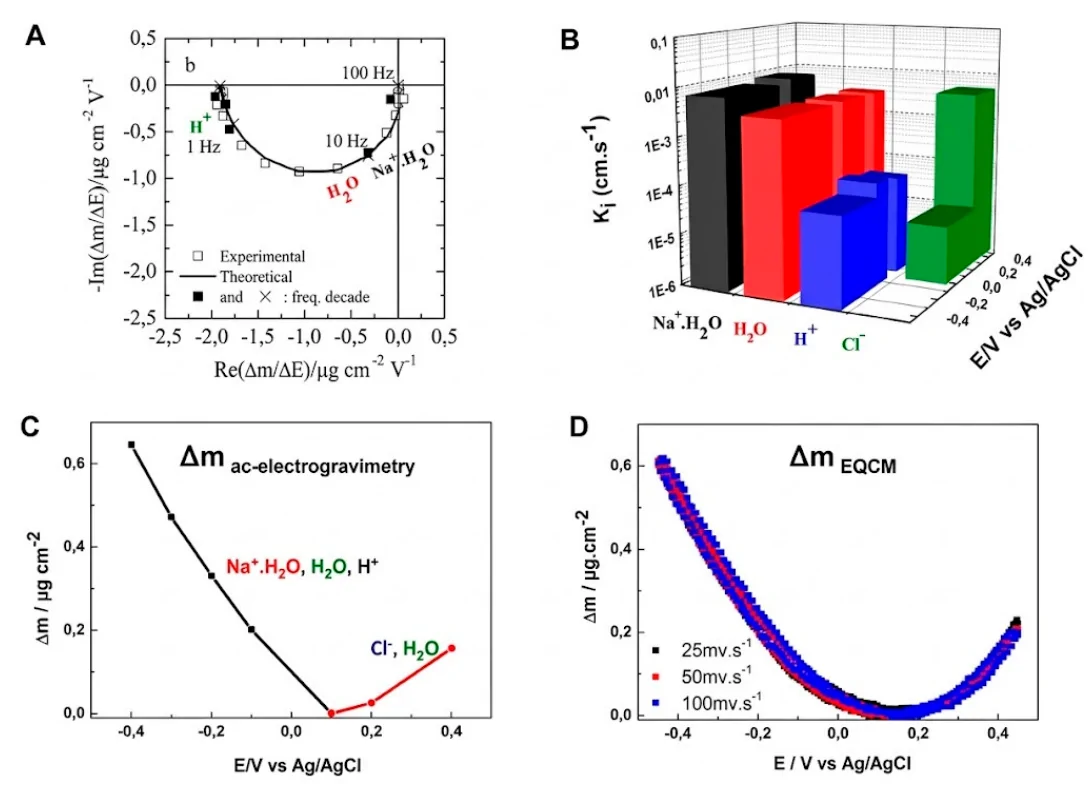
The transfer function, $\frac{\Delta m}{\Delta E}\left(\omega \right)$, at −0.4 V vs. Ag/AgCl (**A**). The kinetic parameters K_i_ (cm.s^−1^) for each ion at different applied potentials (**B**). Total mass variation reconstructed from ac-electrogravimetry (**C**) is compared with the EQCM data (**D**). Reproduced from Ref. [20] with permission from Elsevier (copyright 2016).

**Figure 15:**
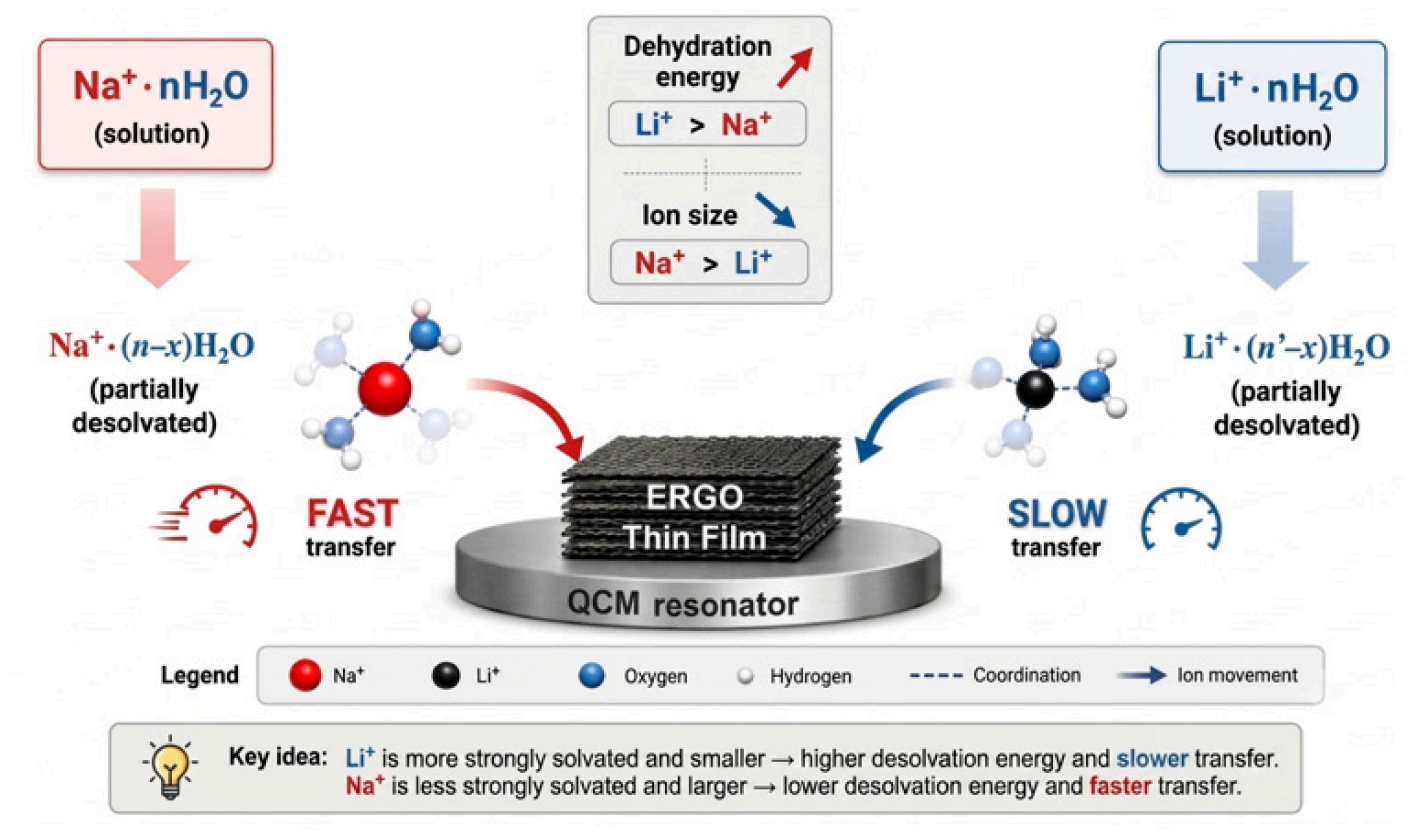
Schematic representation of cation-size-dependent interfacial phenomena at the ERGO/electrolyte interface. Red and blue pathways represent Na^+^ (fast transfer) and Li^+^ (slow transfer) species, respectively. Redrawn and modified based on Ref. [79] with permission from the American Chemical Society (copyright 2017).

**Figure 16:**
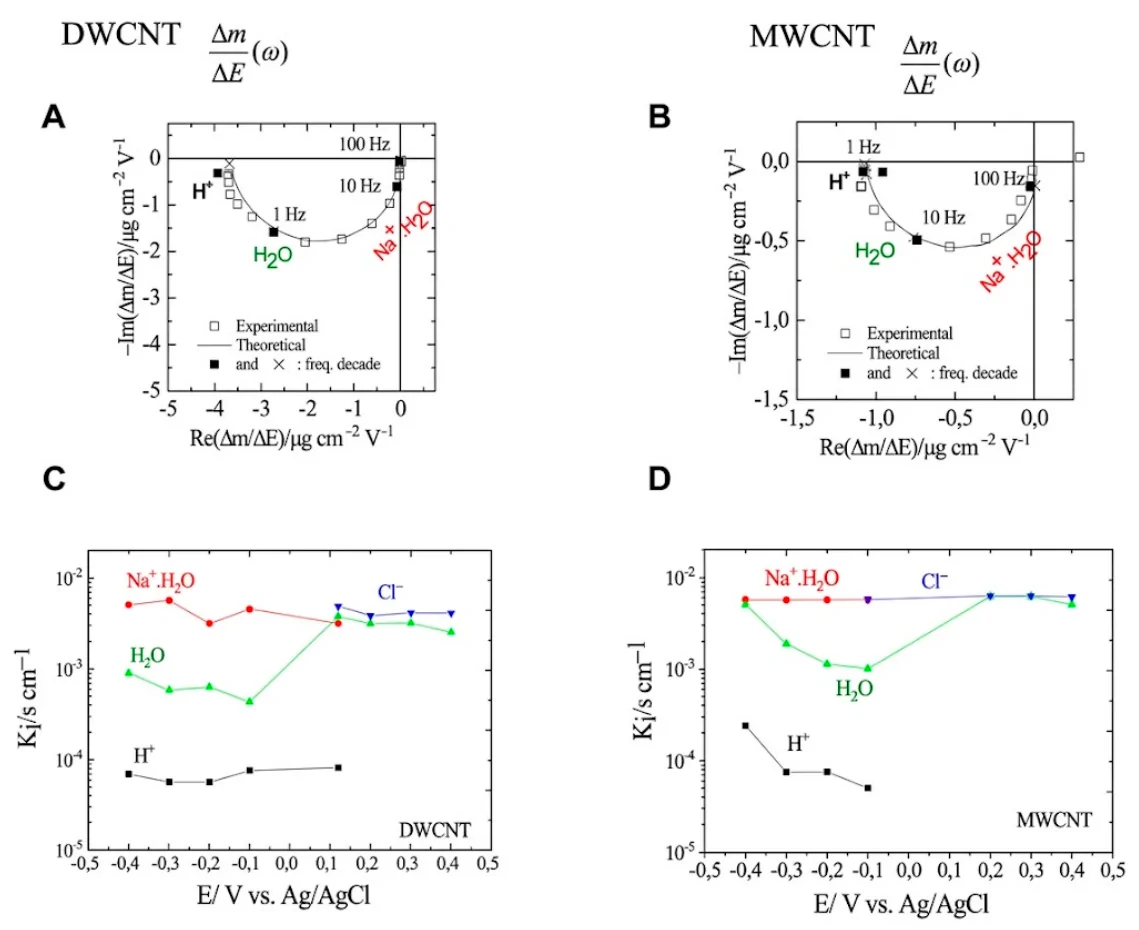
Experimental and fitted ac-electrogravimetric, $\frac{∆m}{∆E}\left(\omega \right)$, profiles for (**A**) DWCNT and (**B**) MWCNT thin-film electrodes. (**C**,**D**) Interfacial transfer kinetic constants (*K_i_*) extracted from model fitting for DWCNTs and MWCNTs, respectively, measured in aqueous NaCl [6]; open access.

**Figure 17:**
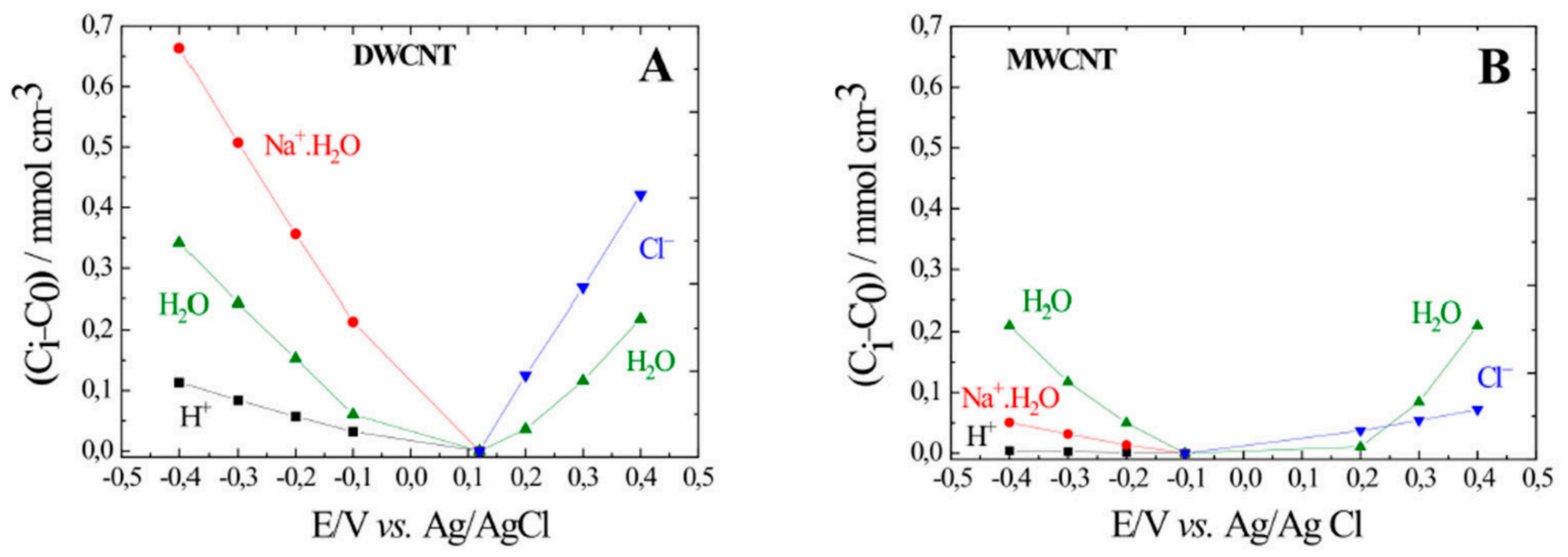
Potential-dependent relative concentration changes (*C_i_* − *C*_0_) of charged and neutral species for (**A**) DWCNT and (**B**) MWCNT film electrodes, measured in aqueous NaCl electrolyte [6]; open access.

**Figure 18:**
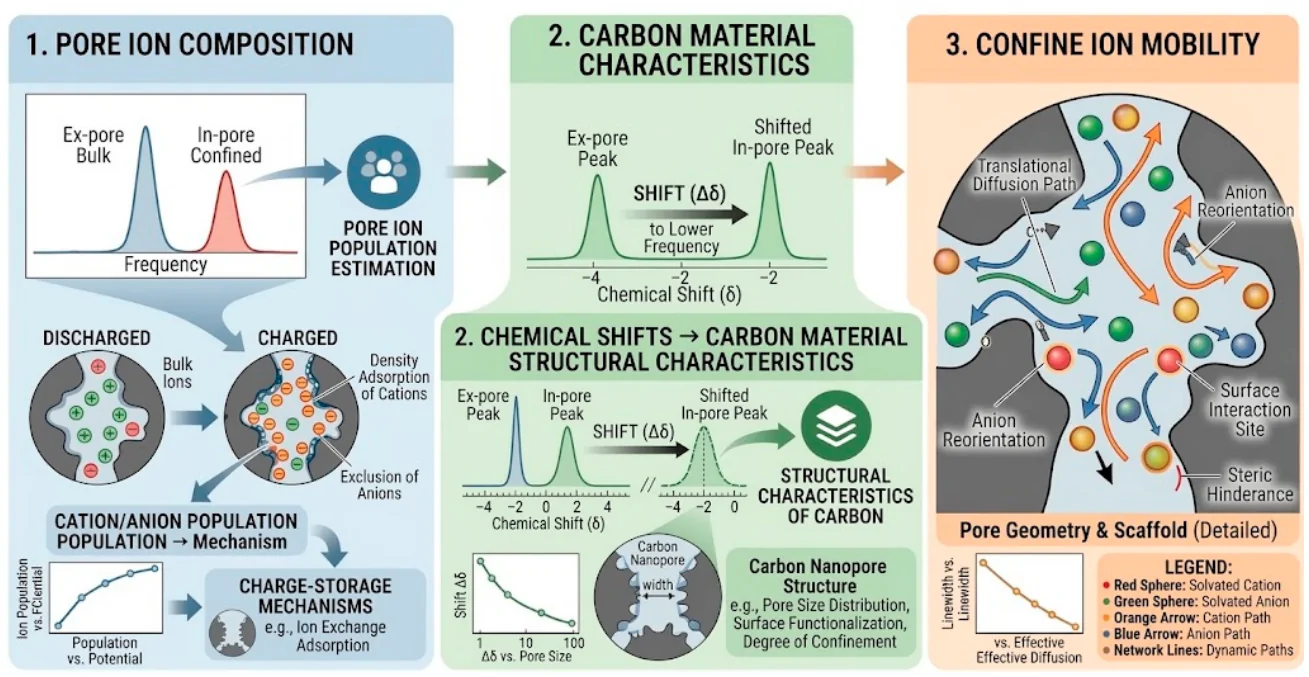
Conceptual schematic illustrating key information pathways derived from NMR spectral parameters in supercapacitor electrodes. (1) Pore Ion Composition: Quantification of bulk (ex-pore) and confined (in-pore) ion populations using resonance peaks and corresponding charge-storage population curves. (2) Carbon Material Characteristics: Relationship between chemical shift displacement (Δ*δ*) to lower frequencies and structural features, including pore size distribution and degree of confinement. (3) Confine Ion Mobility: Dynamic behavior and transport pathways within the nanoporous carbon scaffold geometry.

**Figure 19:**
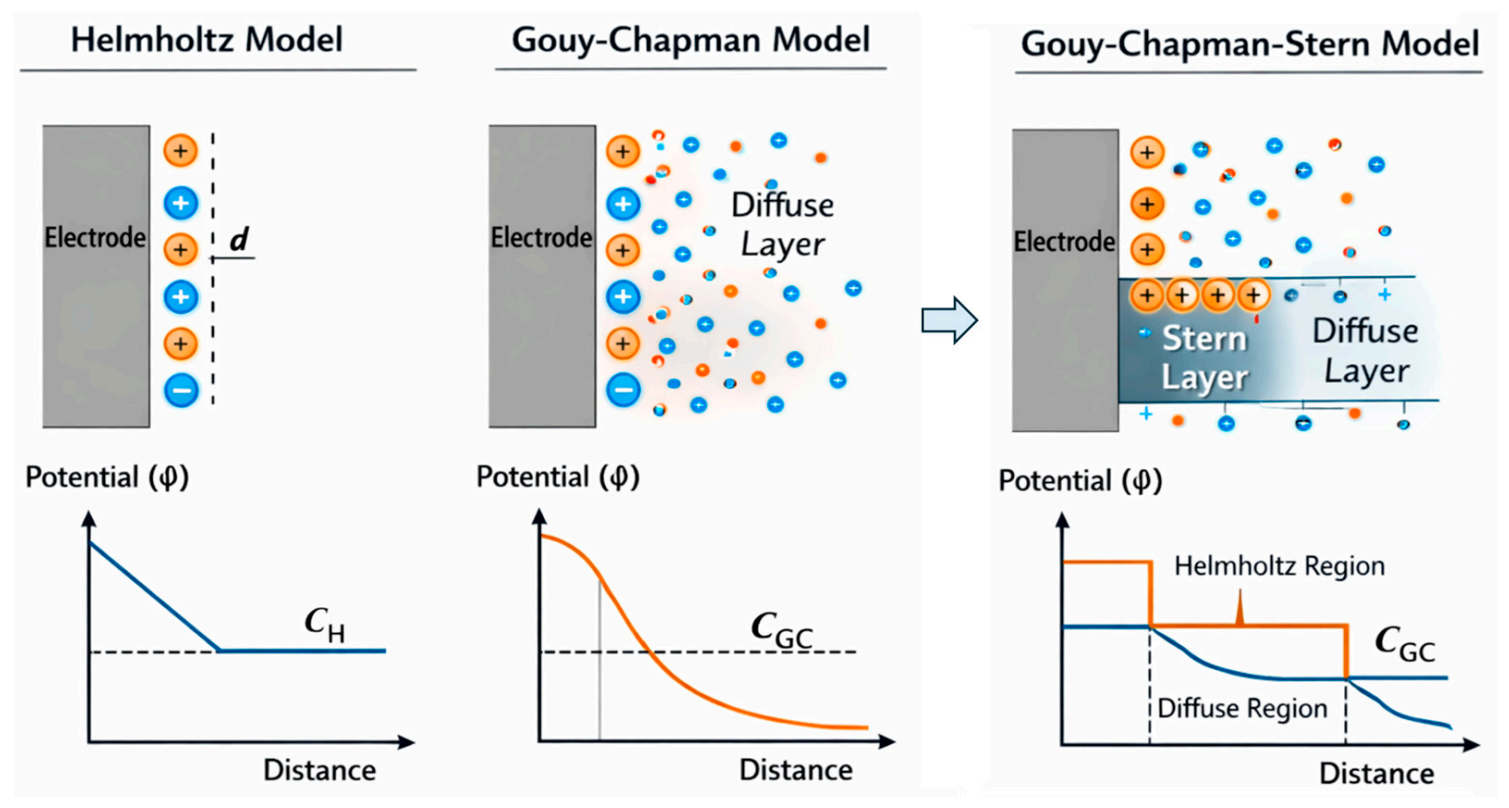
Schematic view of Gouy–Chapman–Stern (GCS). Schematic view of the electric double layer (EDL) models (Helmholtz, Gouy–Chapman, and Gouy–Chapman–Stern) and their potential (*ϕ*) profiles vs. distance. Orange/blue spheres denote cations (+) and anions (−), respectively. Solid blue and orange lines show linear and exponential potential decays across the compact Helmholtz (*C_H_*) and diffuse (*C_GC_*) regions.

**Table 1:** Critical analysis of key diagnostic and simulation tools.

Diagnostic Tool	Primary Information Obtained	Main Strengths	Critical Limitations
Electrochemical impedance spectroscopy (EIS)	Frequency-dependent impedance; resistance, relaxation, charge-transfer, and transport characteristics	Highly sensitive to kinetic and transport processes over multiple timescales	Equivalent-circuit fitting is often non-unique; circuit elements may lack unique physical meaning; porous electrodes rarely behave like ideal capacitors
EQCM/EQCM-D	Potential-dependent mass and dissipation changes	Directly couples electrochemical charge with mass transport; EQCM-D additionally probes viscoelastic effects	Sauerbrey interpretation can fail for porous/soft films; solvent coupling and viscoelasticity may complicate mass assignment
Ac-electrogravimetry	Frequency-dependent charge–mass transfer functions and kinetic parameters	-Disentangles individual species (e.g., Na^+^or solvated Na^+^, H_2_O, H^+^). -Separates fast vs. slow transfer kinetics	-Complex mathematical deconvolution and fitting requirements. -Requires ultra-thin, rigid electroactive films
NMR spectroscopy	Chemical environment, ion coordination, mobility, diffusion, relaxation, population changes	Provides molecular-level information on electrolyte species	Signal overlap, sensitivity, and interpretation of confined or heterogeneous environments can be challenging; operando measurements are technically demanding
Computer Simulations (MD, DFT)	Atomic-scale ion distributions, electrode charge polarization, and pore confinement dynamics.	-Provides full molecular resolution of interfacial ion restructuring. -Isolates key physical parameters without background experimental noise.	-High computational cost when modeling complex or hierarchical pore networks. -Standard force fields fail to capture true faradaic electron transfer in pseudocapacitive systems.

## Data Availability

No new data were created or analyzed in this study. Data sharing is not applicable to this article.
